# NBEAL2 is required for neutrophil and NK cell function and pathogen defense

**DOI:** 10.1172/JCI91684

**Published:** 2017-08-07

**Authors:** John M. Sowerby, David C. Thomas, Simon Clare, Marion Espéli, Jose A. Guerrero, Kim Hoenderdos, Katherine Harcourt, Morgan Marsden, Juneid Abdul-Karim, Mathew Clement, Robin Antrobus, Yagnesh Umrania, Philippa R. Barton, Shaun M. Flint, Jatinder K. Juss, Alison M. Condliffe, Paul A. Lyons, Ian R. Humphreys, Edwin R. Chilvers, Willem H. Ouwehand, Gordon Dougan, Kenneth G.C. Smith

**Affiliations:** 1 Department of Medicine, University of Cambridge School of Clinical Medicine, Cambridge Biomedical Campus, Cambridge, United Kingdom.; 2 Wellcome Trust Sanger Institute, Wellcome Trust Genome Campus, Hinxton, United Kingdom.; 3 INSERM UMR-996, Inflammation, Chemokines and Immunopathology, Université Paris-Sud, Université Paris-Saclay, Clamart, France.; 4 Department of Haematology, University of Cambridge, Cambridge Biomedical Campus, Cambridge, United Kingdom.; 5 NHS Blood and Transplant, Cambridge Biomedical Campus, Cambridge, United Kingdom.; 6 Division of Infection and Immunity, Cardiff University, Cardiff, United Kingdom.; 7 Cambridge Institute for Medical Research, Cambridge Biomedical Campus, Cambridge, United Kingdom.

**Keywords:** Immunology, Infectious disease, Innate immunity, NK cells, Neutrophils

## Abstract

Mutations in the human *NBEAL2* gene cause gray platelet syndrome (GPS), a bleeding diathesis characterized by a lack of α granules in platelets. The functions of the NBEAL2 protein have not been explored outside platelet biology, but there are reports of increased frequency of infection and abnormal neutrophil morphology in patients with GPS. We therefore investigated the role of NBEAL2 in immunity by analyzing the phenotype of *Nbeal2*-deficient mice. We found profound abnormalities in the *Nbeal2*-deficient immune system, particularly in the function of neutrophils and NK cells. Phenotyping of *Nbeal2*-deficient neutrophils showed a severe reduction in granule contents across all granule subsets. Despite this, *Nbeal2*-deficient neutrophils had an enhanced phagocyte respiratory burst relative to *Nbeal2*-expressing neutrophils. This respiratory burst was associated with increased expression of cytosolic components of the NADPH oxidase complex. *Nbeal2*-deficient NK cells were also dysfunctional and showed reduced degranulation. These abnormalities were associated with increased susceptibility to both bacterial (*Staphylococcus aureus*) and viral (murine CMV) infection in vivo. These results define an essential role for NBEAL2 in mammalian immunity.

## Introduction

*NBEAL2* has been identified as the causative gene in gray platelet syndrome (GPS), a rare autosomal recessive disease characterized by platelet α-granule deficiency, which manifests clinically with thrombocytopenia, agranular platelets, bleeding tendencies, and myelofibrosis (1–3). Recent studies have associated *Nbeal2* deficiency with abnormal megakaryocyte differentiation, platelet formation, and α-granule biogenesis or retention (4–6). NBEAL2 is a member of the BEACH domain–containing protein (BDCP) family. BEACH (beige and Chediak-Higashi) is a conserved domain of approximately 280 residues found in 9 human BDCPs (7). BDCPs are large, multi-domain scaffolding proteins that function through unknown mechanisms. A unifying feature of all BDCPs seems to be their involvement in membrane fission and fusion events that have an impact on granule biogenesis, lysosome function, and autophagy, among other cellular processes (7). Indeed, other BDCPs have been implicated in monogenic immunodeficiencies (8–11).

Of the known GPS patients, 31 have mutations in NBEAL2. Four of these patients show evidence of abnormal susceptibility to infection (12–15), but a lack of comprehensive clinical data means this almost certainly represents an underestimate of the prevalence of immunological abnormalities in NBEAL2 deficiency. Consistent with this, neutrophil granule abnormalities have been described in some patients with GPS (12, 16). We therefore used an *Nbeal2*-deficient mouse (4) to investigate the role of NBEAL2 in immunity.

## Results and Discussion

**Figure 1:**
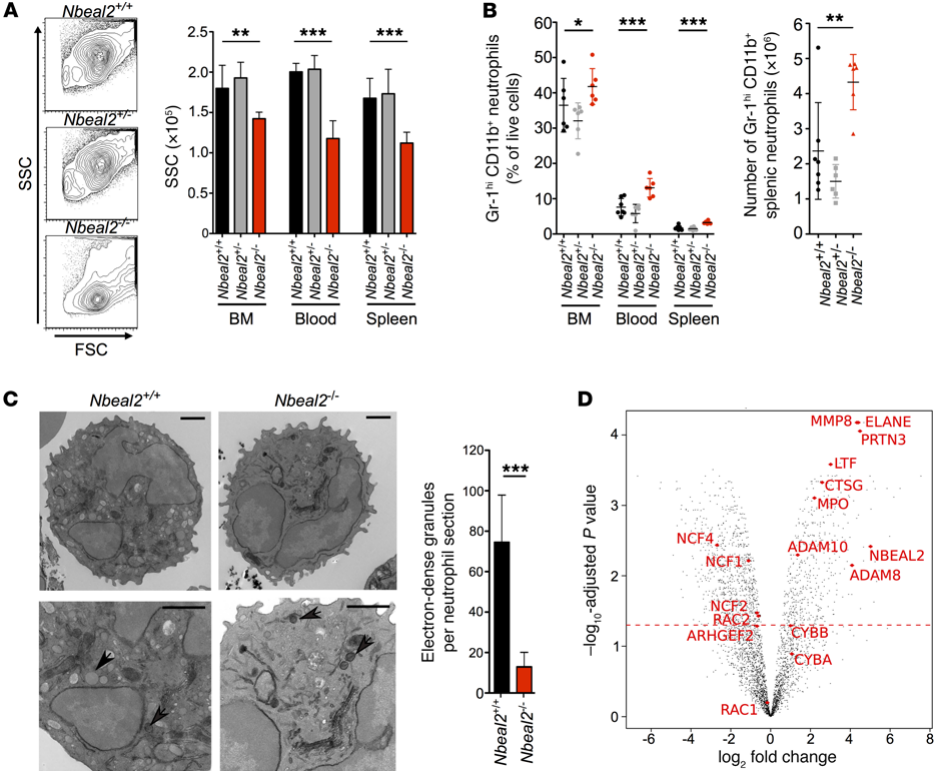
Immunophenotyping shows reduced granularity in *Nbeal2^–/–^* granulocytes. (**A**) Flow plots of BM neutrophils showing forward scatter (FSC) and side scatter (SSC), together with the geometric mean of SSC in Gr-1^hi^CD11b^+^ neutrophils from BM, blood, and spleen (*n* = 6–7). (**B**) Proportion of neutrophils in the BM, blood, and spleen and absolute numbers of splenic neutrophils (*n* = 6–7). (**C**) Transmission electron micrographs of BM neutrophils from whole BM sections. Representative images of neutrophils (top) (original magnification, ×3,500) and a section from the image (bottom) (original magnification, ×6,500). Scale bars: 500 nm (all images). Electron-dense granules (arrowheads) were counted by an investigator blinded to genotype for the WT cells (*n* = 13) and *Nbeal2*^–/–^ cells (*n* = 23) across 3 biological replicates. (**D**) Volcano plot shows the 3,485 proteins identified in the BM neutrophil proteome of *Nbeal2^+/+^* and *Nbeal2*^–/–^ mice. The *y* axis shows FDR-corrected *P* values, the *x* axis displays the fold change (log_2_) of *Nbeal2^+/+^* protein expression compared with *Nbeal2*^–/–^ expression, and the horizontal line shows the cutoff at *P* = 0.05. Granule components, NADPH oxidase subunits, and *Nbeal2* are indicated in red. Error bars indicate the mean and SD. Data are representative of 2 independent experiments (**A** and **B**) or a pooled analysis from 3 independent MS runs (**D**). *P < 0.05, **P < 0.01, and ***P < 0.001, by Kruskal-Wallis (**A** and **B**) and Mann-Whitney *U* (**C**) tests.

Using public RNA expression libraries, we found that *Nbeal2* is expressed highly in the immune system (Supplemental Figure 1A; supplemental material available online with this article; https://doi.org/10.1172/JCI91684DS1). Within the human and mouse immune systems, *Nbeal2* is expressed most highly in neutrophils and NK cells, which, like platelets, are highly dependent on granule exocytosis for their normal function (Supplemental Figure 1, B and C). We therefore examined the granulocytes of *Nbeal2*-KO mice and observed by flow cytometry reduced side-scatter in *Nbeal2^–/–^* neutrophils (Figure 1A). Mixed BM–chimeric mice showed that this observation was neutrophil intrinsic (Supplemental Figure 2). Low side-scatter was also evident in splenic eosinophils (Supplemental Figure 3A). We found that neutrophil numbers were also increased in the BM, blood, and spleens of *Nbeal2^–/–^* mice (Figure 1B). Consistent with this, we observed a reduction in electron-dense granules in *Nbeal2^–/–^* neutrophils (Figure 1C). Neutrophil granules can be divided into 3 types (primary, secondary, and tertiary/gelatinase) depending on protein content and the order of release and synthesis. CD11b, a secondary granule membrane component, was increased on the surface of *Nbeal2^–/–^* BM neutrophils, suggesting a dysregulation of granule exocytosis (Supplemental Figure 3B). These findings were consistent with previous observations in human GPS neutrophils showing reduced granularity via electron microscopy and higher CD11b expression on neutrophils from members of a GPS-affected family (12, 17).

We next assessed whether a lack of visible granules in *Nbeal2*^–/–^ neutrophils corresponded with deficiency, rather than mislocalization, of granule proteins by assessing the whole neutrophil proteome using label-free mass spectrometry (MS) (Figure 1D). We found that 1,290 proteins were differentially expressed between *Nbeal2*^+/+^ and *Nbeal2*^–/–^ neutrophils (Supplemental Table 1). Many major primary granule components, including myeloperoxidase, elastase, proteinase 3, cathepsin G, and CD63 were detected at much lower levels in *Nbeal2*-deficient neutrophils, as were secondary granule components such as lactoferrin, matrix metalloproteinase 8, and Rab27A (Supplemental Table 1). Protein set enrichment analysis of Rab and other GTPase family members revealed a relative reduction in Rab family members (Supplemental Figure 4A), which control many critical aspects of vesicle biology. These differences could not be accounted for by differences in mRNA expression (Supplemental Table 2). Protein ontology analysis of proteins downregulated by 2.5-fold (log_2_) in *Nbeal2*^–/–^ neutrophils revealed significant enrichment of granule- and vesicle-associated protein terms (Supplemental Table 3). To confirm that the downregulated proteins were enriched for granule components, we performed protein set enrichment analysis using lists of proteins identified in human neutrophil granule subsets (18). We found significant enrichment of granule (primary, secondary, and gelatinase) and cell membrane proteins within *Nbeal2^+/+^* versus *Nbeal2^–/–^* neutrophils (Supplemental Figure 4), consistent with the absence of multiple granule subsets in the latter.

**Figure 2:**
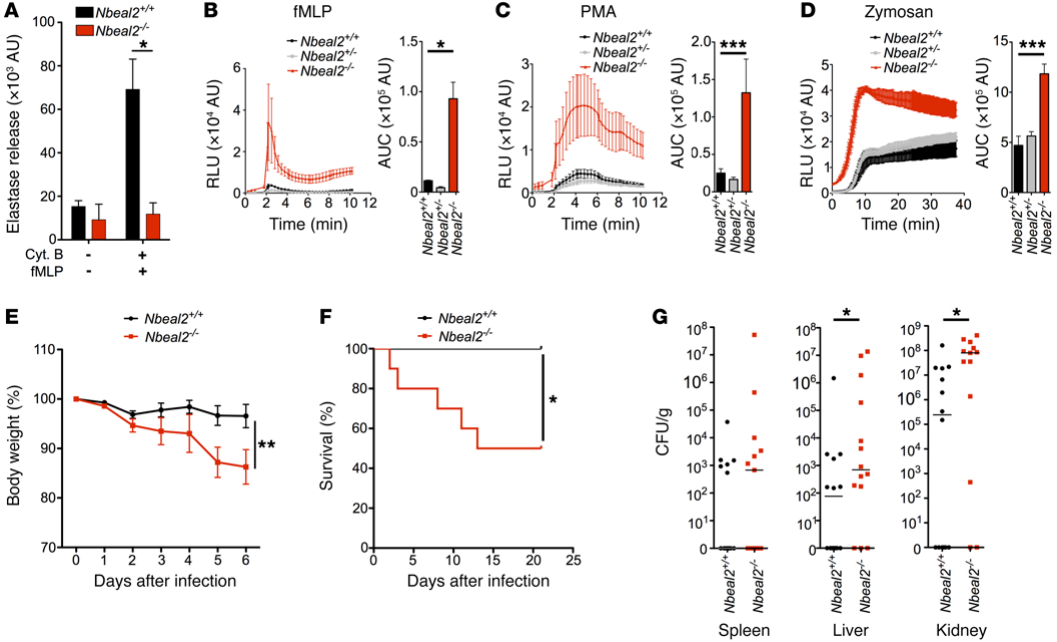
*Nbeal2*^–/–^ neutrophils have altered effector functions and show susceptibility to *S. aureus* in vivo. (**A**) Extracellular elastase release of BM neutrophils in response to cytochalasin B (Cyt. B) and fMLP (*n* = 4). (**B**–**D**) BM neutrophils stimulated with fMLP (**B**), PMA (**C**), or zymosan (**D**) and luminol cleavage fluorescence were quantified over time and the AUC calculated (*n* = 3). (**E**–**G**) Mice were infected i.v. with the sh100 strain of *S*. *aureus* and (**E**) monitored daily for weight changes during the initial 6 days after infection (*n* = 5–7). (**F**) Survival curve of mice infected and monitored for 22 days. Mice were sacrificed if their weight dropped more than 20% from their starting weight (*n* = 9–10). (**G**) Pooled bacterial counts in the kidney, liver, and spleen on day 6 after infection from 2 independent experiments (*n* = 5–7). Data are presented as the mean and SD (**A**), the mean and SEM (**B**–**E**), or the median (**G**). Data are representative of 2 independent experiments. *P < 0.05, **P < 0.01, and ***P < 0.001, by Mann-Whitney *U* test (**A** and **G**), Kruskal-Wallis test (**B**–**D**), 2-way ANOVA (**E**), or log-rank test (**F**). RLU, relative light units.

Consistent with these granule defects, *Nbeal2*-deficient neutrophils released negligible amounts of the primary granule component elastase extracellularly following stimulation with cytochalasin B and formyl-methionyl-leucyl-phenylalanine (fMLP) (Figure 2A) and had no detectable neutrophil elastase by confocal microscopy or Western blotting (Supplemental Figure 5, A and B). Myeloperoxidase (MPO), another major primary granule enzyme, was also reduced in *Nbeal2^–/–^* neutrophils (Supplemental Figure 5B).

In addition to granule proteins, the production of superoxide free radicals by the NADPH oxidase is an important contributor to the antimicrobial action of neutrophils. Gp91^phox^ (CYBB), a membrane-bound component of the NADPH oxidase known to partially localize to secondary granules (19), and the other membrane-bound subunit p22^phox^ (CYBA) were not significantly downregulated in *Nbeal2^–/–^* neutrophils (Supplemental Table 1). Interestingly, the cytosolic components of the NADPH oxidase (NCF1, NCF2, NCF4, and RAC2) were increased in *Nbeal2^–/–^* neutrophils (Figure 1D, Supplemental Table 1, and Supplemental Figure 5C).

*Nbeal2*-deficient neutrophils generated increased ROS in response to soluble ROS-inducing agonists, such as fMLP and PMA, or opsonized particulate zymosan, whether detected by chemiluminescent luminol (Figure 2, B–D) or lucigenin (Supplemental Figure 5D). Several factors may contribute to this enhanced phagocyte respiratory burst. First, generation of superoxide is enhanced in the absence of MPO, which is only expressed at very low levels in *Nbeal2*^–/–^ mice (Supplemental Figure 5B) (20). Neutrophils treated with chemical inhibitors of MPO or those from MPO-deficient individuals produce increased superoxide in response to several stimuli (21). Second, increasing the availability of cytoplasmic subunits such as p47 and p67 increases the generation of ROS (22, 23), and we found these subunits to be increased in NBEAL2 deficiency. Third, an increased oxidative burst could, in theory, result from abnormal localization of the membrane-bound Gp91^phox^ and p22^phox^ that might occur in the absence of secondary granules in *Nbeal2*^–/–^ neutrophils.

Given the granule deficiency of *Nbeal2*^–/–^ neutrophils and that case reports have identified an altered neutrophil phenotype and recurrent infections in humans with *NBEAL2* deficiency, we investigated whether *Nbeal2*-deficient mice have increased susceptibility to infection in vivo. We first challenged *Nbeal2^–/–^* mice with *Staphylococcus aureus* using the sh1000 strain, as neutrophils are essential for immunity against this pathogen (24). *Nbeal2*^+/+^ mice typically experienced a mild illness, but 50% to 60% of *Nbeal2*^–/–^ mice developed weight loss necessitating euthanasia within 6 days of infection (Figure 2, E and F). This was associated with increased bacterial counts in the kidney and liver (Figure 2G) as well as histological evidence of severe uncontrolled infection in *Nbeal2*^–/–^ kidneys, with microabscesses, neutrophil infiltrates, acute tubular necrosis, and areas of infarction (Supplemental Figure 6A). In vitro, *Nbeal2*^–/–^ neutrophils showed intact phagocytosis of *S*. *aureus*, as well as an augmented phagocyte respiratory burst against opsonized *S*. *aureus* (Supplemental Figure 6, B and C). To determine the role of neutrophils in the NBEAL2 deficiency phenotype, we compared *S. aureus* infections in neutrophil-depleted *Nbeal2*^+/+^ and *Nbeal2*^–/–^ mice. Depletion of neutrophils in *Nbeal2*^+/+^ mice led to increased bacterial burden in the kidney (a site in which a lack of bacterial control has been associated with overwhelming sepsis and mortality [ref. 25]), comparable to that seen in isotype-treated *Nbeal2*^–/–^ mice. This observation, together with the relative lack of impact of neutrophil depletion on *Nbeal2*^–/–^ mice, suggested that the dominant cause of the staphylococcal susceptibility in *Nbeal2*^–/–^ mice is NBEAL2 deficiency in neutrophils (Supplemental Figure 6D).

**Figure 3:**
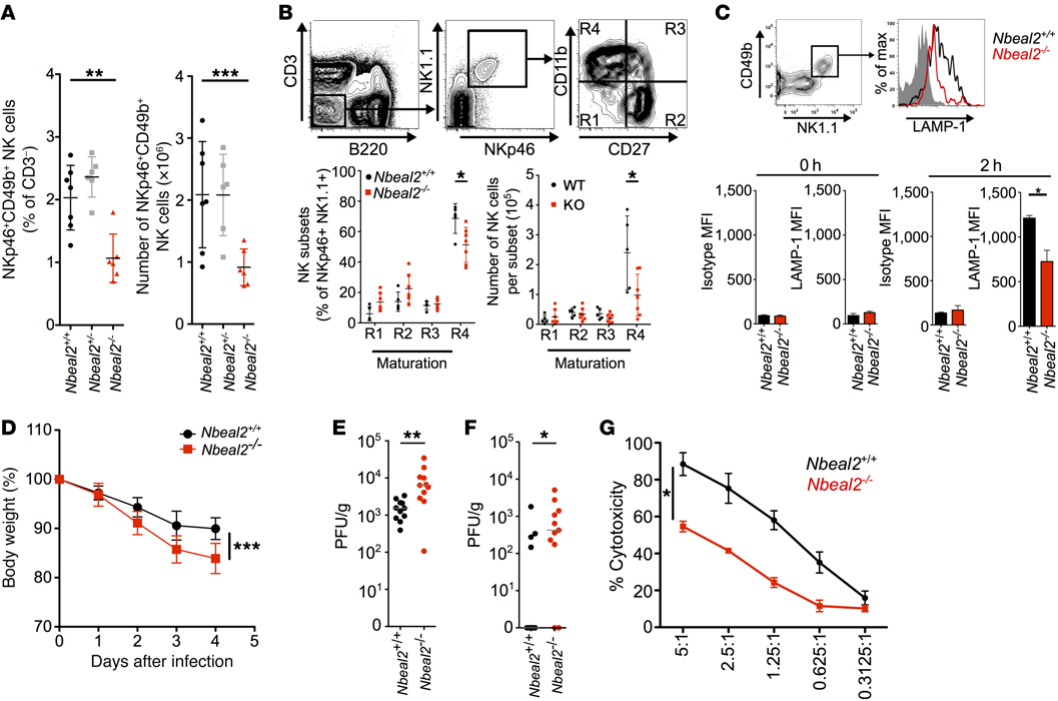
*Nbeal2^–/–^* mice have dysfunctional NK cells in vitro and an impaired response to mCMV in vivo. (**A**) Proportion and absolute numbers of CD3^–^B220^–^CD49b^+^NKp46^+^ splenic NK cells for the indicated genotypes. (**B**) Expression of CD11b and CD27 (maturation markers) on CD3^–^B220^–^NK1.1^+^NKp46^+^ NK cells. Maturation was measured from gates R1 to R4 (with R4 being the most mature). Shown are the proportions and absolute numbers for each subset (*n* = 5–7). (**C**) Surface expression of LAMP-1/isotype of splenic NK cells 0 and 2 hours after PMA/ionomycin stimulation. Representative FACS gating and LAMP-1 histogram showing isotype control antibody staining (gray area) and LAMP-1 in *Nbeal2^+/+^* (black line) and *Nbeal2^–/–^* mice (red line) (*n* = 4–5). max, maximum; MFI, mean fluorescence intensity. (**D**–**F**) *Nbeal2^+/+^* or *Nbeal2^–/–^* mice were infected (i.p.) with 3 × 10^4^ salivary gland–propagated Smith strain mCMV, and BW was monitored for 4 days after infection (**D**). On day 4, mice were sacrificed, and virus PFU were quantified in the spleen (**E)** and lungs (**F**) (*n* = 11). (**G**) Splenic NK cells were isolated and cultured in 1,000 U IL-2 for 4 days before cytotoxicity (LDH release) was tested on YAC-1 cells (*n* = 3). Data are presented as the mean and SD (**A**–**D**), median (**E** and **F**), or SEM (**G**) and are representative of 3 (**A**, **E**, and **F**) or 2 (**B**, **C**, and **G**) independent experiments. *P < 0.05, **P < 0.01, and ***P < 0.001, by Kruskal-Wallis test (**A**), Mann-Whitney *U* test (**B**, **C**, **E**, and **F**), or 2-way ANOVA (**D** and **G**).

Given that NK cells also rely on perforin- and granzyme-containing granules to control viral infections, we reasoned that antiviral immunity might be affected in *Nbeal2^–/–^* mice. The number of splenic NK cells was reduced in *Nbeal2^–/–^* mice (Figure 3A), with the major abnormality being reduced numbers in the mature NK cell subset (CD11b^+^, CD27^–^) (Figure 3B). We assessed NK cell degranulation in vitro by measuring externalized lysosome-associated membrane protein 1 (LAMP-1) following stimulation. Although there was no difference in surface LAMP-1 expression at baseline or in total LAMP-1 expression (Supplemental Figure 7A), *Nbeal2^–/–^* NK cells failed to fully upregulate LAMP-1 after PMA and ionomycin stimulation (Figure 3C). To investigate whether reduced NK cell numbers and degranulation had in vivo consequences, we infected mice with murine CMV (mCMV). *Nbeal2^–/–^* mice became more unwell than did their *Nbeal2*^+/+^ counterparts, with increased weight loss after infection (Figure 3D) and with markedly more viral PFU in the spleen and lungs on day 4 after infection (Figure 3, E and F). This difference was likely due to reduced NK cytotoxicity (Figure 3G) and not CD8 killing, which was grossly normal (Supplemental Figure 7B). Control of mCMV in the liver has been reported to be more dependent on IFN-γ (26), so the unchanged viral PFU observed in the livers of *Nbeal2*^–/–^ mice is consistent with their intact NK cell IFN-γ production (Supplemental Figure 7, C and D).

NK cell depletion is known to increase mCMV titers (e.g., ×10^3^ to ×10^4^ in the spleen; ref. 27), which we confirmed with anti-NK1.1 treatment (splenic PFU increased by 10^4^; Supplemental Figure 7E). The increase in mCMV PFU observed in *Nbeal2*^–/–^ mice (approximately ×10^1^ to ×10^2^ in lung and spleen) was therefore intermediate between those seen in *Nbeal2*^+/+^ and NK-depleted mice (Figure 3, E and F). Consistent with this intermediate phenotype, after weight loss over the first 4 days, the BW of *Nbeal2*^–/–^ mice stabilized, and, by day 14 after infection, salivary gland viral loads were the same as those in *Nbeal2*^+/+^ mice (Supplemental Figure 7F), indicating that *Nbeal2*^–/–^ mice could eventually control infection.

We have demonstrated that *Nbeal2* plays an important role in immunity, as its absence results in an altered development or function of granules in neutrophils and NK cells. The lack of *Nbeal2* in neutrophils led to a marked downregulation of multiple granule proteins and effector molecules. Consistent with the lack of MPO and the increase in cytoplasmic components of the phagocyte respiratory burst, superoxide production was substantially enhanced in *Nbeal2*^–/–^ neutrophils. Despite enhanced superoxide production, *Nbeal2*^–/–^ mice were more susceptible to *S*. *aureus* infection, emphasizing the importance of intact granule function for defense against *Staphylococcus*. Degranulation, cytotoxicity, and maturation were also disrupted in NK cells, and *Nbeal2*-deficient mice showed increased susceptibility to mCMV. In summary, our study defines what to our knowledge is a previously undescribed role for *Nbeal2* in immunity. Detailed investigation of the immune system in patients with *NBEAL2* mutations would extend these findings, and there may be benefit in screening for *NBEAL2* in patients with primary immune deficiencies.

## Methods

Further information can be found in the supplemental material. See complete unedited blots in the supplemental material.

### Statistics.

*P* values were calculated using a 2-tailed Mann-Whitney *U* test, Kruskal-Wallis 1-way ANOVA, 2-way ANOVA, or a log-rank test. *P* values of less than 0.05 were considered statistically significant.

### Study approval.

All in vivo experiments were performed according to the regulations of the UK Home Office Scientific Procedures Act (1986). All studies were reviewed and approved by the University Biomedical Services via the Named Animal Care and Welfare Officer.

## Author contributions

JMS, DCT, ME, IRH, SC, PAL, AMC, ERC, WHO, GD, and KGCS designed and/or organized experiments. JMS, DCT, and ME performed the majority of phenotyping and functional assays. JAG and JMS carried out transmission electron microscopic analyses. IRH, MM, MC, and JAK performed NK functional assays and all mCMV plaque assays. SC and K. Harcourt organized and performed all in vivo infections. JKJ and K. Hoenderdos performed several neutrophil functional assays. RA and YU performed MS analyses. PRB carried out CD8 cytotoxicity assays. PAL and SMF analyzed microarray data. JMS, DCT, and KGCS wrote the manuscript.

## Supplementary Material

Supplemental data

## Version 1 08/07/2017

Electronic publication

## Version 2 09/01/2017

Print issue publication
