# Supplementary material for: NBEAL2 is required for neutrophil and NK cell function and pathogen defense
Source: J Clin Invest. 2017 Aug 7;127(9):3521–6. doi: 10.1172/JCI91684 (PMC5669559; doi:10.1172/JCI91684)

Supplementary Material

Materials and Methods

Mice

All experiments were performed according to the regulations of the UK Home Office Scientific Procedures Act (1986). *Nbeal2*$^{-/-}$ mice were generated as part of as international consortium (www.knockoutmouse.org) and have been reported previously (1). Mice from this pipeline are generated from targeted C57BL/6N embryonic stem cells (2). Mice used were C57BL/6N (also referred to as *Nbeal2*$^{+/+}$ in the text) and C57BL/6 CD45.1 (Central Biomedical Services, University of Cambridge). Adult mice used were between 2-6 months old unless otherwise stated and always with age-matched controls. Mice used for immunisations were between 8-12 weeks old. For the chimera experiments *Nbeal2*$^{-/-}$ mice were first crossed to B6 CD45.1 controls. Heterozygous mice were then bred to obtain CD45.1 fixed mice for either genotype.

Bone marrow chimeras

To generate mixed bone marrow chimeras, C57BL/6 mice were lethally irradiated (1000 Rads) and reconstituted using intravenous injection of 3 x 10^6 bone marrow cells. Bone marrow cells were a 50:50 mixture of CD45.1 and CD45.2 congenically marked cells from *Nbeal2*$^{+/+}$ and *Nbeal2*$^{-/-}$ mice. Chimeric mice received neomycin sulphate in their drinking water for 4 weeks whilst BM was reconstituted. Reconstitution was checked by flow cytometry of whole blood at 6 weeks and mice were used at 6-8 weeks post-irradiation.

Transmission electron microscopy

Whole bone marrow from either *Nbeal2*$^{+/+}$ or *Nbeal2*$^{-/-}$ mice was flushed from the femur and tibia of euthanized mice. Cell pellets were then washed, fixed and analysed as described previously (1). Electron dense granules were enumerated manually, blinded to genotype and analysed in GraphPad.

Neutrophil elastase (NE) degranulation assay

Isolated bone marrow neutrophils were re-suspended phosphate buffered saline (PBS) (with Mg^{2+} and Ca^{2+}) (11.1x10^6/ml). 270 µl aliquots were transferred to 2 ml Eppendorf tubes and incubated at 37°C for 1 h. After 1 hour the cells were primed with cytochalasin B 5 µg/ml (Sigma Aldrich, UK) for 5 min an activated with 10 µM fMLP (Sigma Aldrich) or vehicle for 10 minutes. Cells were pelleted and the supernatant transferred to fresh tubes. Samples were frozen and stored at -80°C until further analysis. Measurement of NE activity was performed using the EnzChek® activity assay (Molecular Probes, Thermo, UK), which is based on the cleavage of DQ-Elastin.

Neutrophil Phagocytosis Assay

PMN phagocytic capacity was assessed using pHrodo™ RED *Staphylococcus aureus* Bioparticle® conjugates (Life Technologies, A10010 and P35361) resuspended in HBSS pH 7.5 (to a final concentration of 1 mg/ml) according to the manufacturer's recommendations. The pHrodo™ *S aureus* Bioparticle® suspension was sonicated to generate a homogenous dispersion prior to incubating with PMNs (10-12 particles/PMN) at 37°C for 30 minutes and then collected for flow cytometry and cytospins. This assay is based on differential fluorescence of the bioparticle in an acidic environment, particles in the extracellular environment or those adherent to the outer surface of the PMN display little or no fluorescence. Fluorescence measured using excitation 530nm/emission 590nm. Internalization was also verified by confocal images of cytospins and live cell confocal imaging. Cytospins were generated as indicated above and prepared using a DAPI containing mountant (Invitrogen)

Elastase Immunofluorescence

Isolated neutrophils were fixed in 3.5% PFA in PBS (with Mg^{2+} and Ca^{2+}) for 20 minutes at room temperature. Cells were then washed briefly three times in PBS before being transferred onto glass slides using Cytospin (Thermo). Cells were permeabilized with 0.5% Triton X-100 (Sigma) in PBS for 10 minutes at room temperature, blocked with 0.5% (w/v) BSA in PBS for 30 minutes, and then incubated with anti-mouse elastase clone M-18 (Santa Cruz Biotechnology) in 0.1% (w/v) BSA in PBS for 1 hour at room temperature. Slides were washed briefly, three times in PBS before being stained with Alexa Fluor 488 donkey anti-goat IgG secondary (Invitrogen) in 0.1% (w/v) BSA in PBS for 1 hour at room temperature. Slides were washed twice in PBS and then stained with rhodamine-phalloidin (Invitrogen) in 0.1% (w/v) BSA in PBS for 20 minutes. Slides were washed twice in PBS then mounted in MOWIOL (Sigma) containing DAPI (Sigma).

Neutrophil Isolation

Mice were sacrificed and bone marrow was flushed from the femurs and tibias with cold PBS supplemented with 0.5% BSA. Cells were washed, and resuspended in cold PBS-BSA before being counted. Neutrophils were then isolated using the neutrophil isolation kit (Miltenyi, UK) following the manufacturer's protocol. Purity was checked by flow cytometric analysis, staining for Gr1 and CD11b.

Transcriptomics

For the analysis of human leukocyte subsets, B cells, T cells, monocytes and neutrophils were separated from the blood of healthy volunteers (with ethical approval from NRES Committee East of England - Cambridge Central (refs: 08/H0308/176, 04/023 and 08/0306/21)) as described previously (2). Briefly, peripheral blood mononuclear cells (PBMC) were isolated via centrifugation over Histopaque 1077 (Sigma) at 800g for 20 minutes, breaks off. Each cell type was isolated using the CD4, CD8, CD19 (B cell) and CD14 (monocyte) microbeads (Miltenyi). CD16 microbeads (Miltenyi) were used to isolate neutrophils from the red cell/granulocyte pellet after the Histopaque spin and red cell lysis treatment. Murine neutrophils were isolated as described in "Neutrophil isolation". Cell pellets were lysed in RLT (Qiagen, UK) and RNA was extracted using the RNEasy Mini Kit (Qiagen) according to the manufacturer's protocol. RNA integrity was tested by capillary electrophoresis using

a BioAnalyser 2100 (Agilent Technologies) and its concentration determined using a NanoDrop ND-1000 spectrophotometer. 200ng of RNA was labelled using the WT Sense Target labelling kits (Affymetrix) and hybridised to Mouse Gene 1.0 ST arrays (mouse samples) or the Human Gene 1.1 ST array (human samples) according to the manufacturer's instructions. After washing, arrays were scanned using either a GS 3000 scanner or a GeneTitan instrument (Affymetrix). The resulting raw microarray data was normalized and summarized using the RMA function of the oligo package in BioConductor. Human samples were hybridised across a number of batches; batch effects were removed using the ComBat algorithm with leukocyte subset as a covariate. Mouse arrays were run on the same batch. Differentially expressed genes were identified using the limma package. Differential expression was defined as fold changes greater than 0.5 fold that were statistically significant following correction for multiple testing by setting the false discovery rate to 5%.

Neutrophil respiratory burst

Isolated neutrophils were resuspended in PBS at a concentration of $1x10^7$ per ml and warmed to 37 °C for 15 minutes. 150µl of neutrophils were incubated for 3 minutes with 2 µM luminol (Sigma) or 100 µM lucigenin (Sigma) and 62.5U/ml horseradish peroxidase. Post-incubation cells were stimulated with 200 ng/ml PMA (Sigma), 16 mM fMLP (Sigma) or 100 µg/ml zymosan (Sigma). For measuring the phagocyte respiratory with S. *aureus* sh1000, bacteria were first grown for 4-6 hours at 37°C in Lysogeny Broth media to reach exponential phase. Bacteria were washed twice in PBS before being resuspending in 50% mouse serum (in PBS) and incubated with rotation for 15 minutes. Bacteria were then washed again in PBS before being resuspended at 6×10^9/ml in PBS. Bacteria were used at a ratio of 20:1 (bacteria:neutrophils). Light emission was measured using a luminometer (Berthold Technologies, UK). fMLP and PMA were added through the injection port. Data was collected and output is in relative light units against time. Graphs were drawn to represent this using 3 biological replicates (or technical where indicated). The area under the curve was obtained using Graphpad Prism and values compared using an unpaired Students t-test.

Western blot of neutrophil lysates

2×10^5 isolated neutrophils aliquots were lysed in 50µl 1X LDS buffer (Invitrogen, UK) containing 10% β-mercaptoethanol (Sigma). Samples were boiled for 10 minutes at 70 °C before equal volume loading into pre-cast NuPAGE Novex 4-12% Bis-Tris gels (Invitrogen). Spectra™ Multicolor Broad Range Protein Ladder (Invitrogen) was used as a size ladder for each gel. Electrophoresis was run using standard conditions before proteins were transferred onto PVDF (Millipore, UK) membrane at 350mA for 1 hour. Membranes were blocked with non-fat dry milk powder (Marvel, UK) in TBST buffer (1X Tris buffered saline with 0.1% (vol/vol) Tween20 (Sigma)) at room temperature for 1 hour. Primary antibodies purchased from Santa Cruz were: anti-gp91 (54.1) and anti-p22 (FL-195). Primary antibodies purchased from R & D were: anti-Myeloperoxidase (392105) and anti-Elastase (887105). Primary antibodies purchased from MerckMillipore were: anti-p40 (07-501), anti-p47 (07-500) and anti-p67 (07-502). Primary antibody purchased from Abcam was polyclonal anti-beta Actin (ab8227). Antibodies were used at 1:1,000 (except anti-beta actin which was used at 1:5,000) and incubated overnight at 4 °C. Post primary antibody incubation, membranes were then washed three times in TBST for 10 minutes on a rocker before being incubated with the relevant HRP-conjugated secondary antibodies (Cell signalling) at 1:10,000 for 45 minutes to 1 hour at room temperature. Membranes

were then washed again (three times) and once in PBS before being developed with Pierce™ ECL Western Blotting Substrate (Thermo) or SuperSignal™ West Femto Maximum Sensitivity Substrate as per the manufacturer's protocol. The membranes were imaged using the GelDoc XR (Bio-Rad, UK) and semi-quantitative analysis was carried out using the Image Lab software (Bio-Rad).

Mass spectrometry

Neutrophils were isolated as mentioned previously, washed three times in ice-cold PBS and then lysed in 6 M Urea (Sigma) lysis buffer (6 M Urea, 20 mM $MgCl_2$). DNA removed by benzonase (Thermo) treatment following manufacturer's protocol. Protein concentrations were determined using the BCA test (Thermo) and 30µg of protein was sent for MS analysis. Samples were resolved by SDS-PAGE, reduced, alkylated and digested in-gel using trypsin. The resulting peptides were analysed by LC-MSMS using a Q Exactive (Thermo) coupled to an RSLC3000 UHPLC (Thermo). Replicate runs were processed using Maxquant 1.5.2.8 to search a Mus musculus Uniprot database (downloaded 04/11/14) with protein N-terminal acetylation, oxidation (M) and deamidation (N/Q) as variable modifications and carbamidomethylation (C) as a fixed modification. Label free quantitation and Match between runs were enabled and the resulting data was processed in Perseus 1.5.1.6. Reverse and potential contaminant proteins were removed and LFQ intensities log_2 transformed. Data was filtered so proteins require a minimum of 3 intensity values and missing values replaced from a normal distribution. Differentially expressed proteins were identified using the limma package in the R programming environment with changes relative to control neutrophils.

Proteome analysis

For analysis of subcellular location of differentially expressed proteins (DEPs), the limma generated DEPs were first annotated with known or predicted cellular location using the UniProt database (http://www.uniprot.org/uploadlists/). The DEP list was then grouped based on subcellular location/organelle for both upregulated and downregulated proteins. Proteins with more than one localisation entry were grouped based on the additional 'note' information included with the UniProt subcellular location annotation. For example, a protein described as: "cytoplasmic unless phosphorylated post-activation and then translocated to the nucleus" would be grouped as "cytoplasmic related". For PANTHER Gene Ontology Enrichment, significantly downregulated (positive fold-change) proteins in *Nbeal2*$^{-/-}$ neutrophils were filtered based on the log-fold change (≥2.5-fold). Unique proteins were then analysed using the PANTHER Overrepresentation Test (release 20160715) via the online analysis tool (http://geneontology.org/page/go-enrichment-analysis). All filtered proteins identified in the MS analysis were used as the reference for the analysis. Each list was tested against the GO Ontology database (release 2015-10-27) for enrichment of "cellular components" and/or "biological processes". For protein set enrichment, the filtered and normalised MS expression set was analysed using the java based GSEA software (http://www.broad.mit.edu/gsea/). Custom protein sets were designed following the GSEA instructions using the dataset referenced earlier in the text. GSEA was run under default settings with the exception of 'Permutation type' (gene_set) and 'Number of permutations' (10,000). For analysis of GTP family enrichment, a GTPase list was downloaded from the qiagen resource website (https://www.qiagen.com/us/resources/resourcedetail?id=1cff0638-82a2-4003-a86a-bebeadc448f8&lang=en). This list was filtered to remove any GTPases not expressed in our proteome

data and then further subdivided into GTPase families. These lists were then used for protein set enrichment as described above.

Processing of murine tissues

6-10 week old mice were sacrificed and whole blood, spleen and hind legs were collected. Spleens were weighed before being processed by manual dissociation, red blood cell (RBC) lysis treated, washed in FACS buffer (PBS 0.5% BSA) and enumerated. Bone marrow prepared by first removing excess muscle tissue from both the femurs and tibias of sacrificed mice. Bone marrow cells were then flushed from each bone using a 26G needle (BD Bioscience, UK) and 5ml of cold PBS. Cells were RBC lysis treated, washed and enumerated. Blood was collected in clean 1.5 ml centrifuge tubes and allowed to clot for 1 hour on the bench at room temperature. Coagulated blood was spun at 1500g for 15 minutes and serum was collected and stored at -20 °C until needed.

Antibodies and flow cytometry

The following antibodies were purchased from eBioscience (UK): Gr-1 (RB6-8C5), CD11b (M1/70), B220 (RA3-6B2), NK1.1 (PK136), NKp46 (29A1.4), CD21/35 (4E3), CD49b (DX5), CD45.1 (A20), CD45.2 (104), CD23 (B3B4), LAMP-1 (1D4B) and Rat IgG2a K Isotype Control (eBR2a). The following antibodies were purchased from BD Biosciences: SiglecF (E50-2440) and CD16/32 Fc receptor block (2.4G2). The following antibody was purchased from Biolegend: CD3e (17A2). Single cell suspensions were stained in 5ml polystyrene test tubes (BD) and unless otherwise indicated stained and washed with FACS buffer. Initially cells were stained with a live/dead Aqua discrimination dye (Invitrogen) following the manufacturer's instructions. Cells were then washed and stained with antibodies at a dilution of 1:200 with the exception of: CD11b (1:500), Gr-1 (1:500), B220 (1:400) and NKp46 (1:100). Cells were stained for 45 minutes at 4°C before being washed in PBS and fixed in PBS with 2% paraformaldehyde solution. For intracellular staining, extracellular surface staining was carried out as described above. After the final wash step, cells were fixed and permeabilized using the FoxP3 staining buffer set (eBioscience) following the manufacturer's protocol. Cells were then washed and stained with intracellular antibody for at least 1 hour at 4 °C. Cells were then washed and fixed as described before. For detection of IFNγ secreting NK cells, tissues were processed as described previously (3). Briefly, splenocytes from MCMV infected mice were stimulated with PMA (50ng/ml) and ionomycin (500ng/ml) (both Sigma) in the presence of brefeldin A (Sigma) for 5 hours, before extra- and intra-cellular staining. Cells were stained with anti-IFNγ (clone XMG1.2, Biolegend). FACS analysis was performed on a Fortessa II (BD), and data were analyzed with FlowJo software (Tree Star).

NK *in vitro* degranulation assay

For the in vitro degranulation of NK cells a previously described protocol was used(4). In brief, splenocytes prepared as before were counted and resuspended at a concentration of 2 x 10^6 per ml in RPMI containing anti-LAMP-1-PE or isotype antibody conjugated to PE at a dilution of 1:200. 1 x 10^6 splenocytes were plated into wells of a 24-well plate and stimulated with 100 /ml PMA and 150ng/ml ionomycin (both Sigma) for indicated time points. Post-stimulation cells were retrieved from wells, washed and processed for extracellular staining as described earlier.

T cell Cytotoxcity assay

Single cell suspensions of splenocytes from *Nbeal2*$^{+/+}$ and *Nbeal2*$^{-/-}$ mice were made, before being enumerated. Splenocytes were plated in 6-well plates pre-coated with anti-CD3e (1µg/ml) and anti-CD28 (2µg/ml) at a density of 1.7 × 10^6/ml (8ml total) in T cell media (RPMI (Sigma) plus 10% fetal calf serum (Sigma), 10U/ml Penicillin Streptomycin (Thermo), 1mM Sodium Pyruvate (Sigma), 2mM L-glutamine (Sigma), 50µM 2-Mercaptoethanol and IL-2 (20ng/ml). 48 hours post-stimulation cells were washed, resuspend in 20ml T cell media and 5ml plated per well of a 6-well plate. On day 6 cells were used for cytotoxicity assay. The target cell line P815 (ATCC, UK) was cultured in DMEM (Sigma) with 10% fetal calf serum and split one day prior to assay being carried out. On the day of the assay P815 were washed, and counted, and loaded with anti-mouse CD3e (clone 145-2c11, eBioscience). Cytotoxic T cells were counted, washed, resuspended media plus 1% fetal calf serum before being plated in a U-bottomed 96-well plate in triplicate per condition/genotype. A starting effector:target ratio of 10 was used and diluted serially. 10^4 P815 cells were added to each plate as targets. No target controls were included for each dilution, as well as media alone, targets alone, targets with lysis buffer and media with lysis buffer. The lysis buffer was part of the lactate dehydrogenase (LDH) release kit (Promega, UK), which was used to quantify target cell lysis following the manufacturers instructions.

NK Cytotoxicity assay

Single cell suspensions of splenocytes from *Nbeal2*$^{+/+}$ and *Nbeal2*$^{-/-}$ mice were made, before being enumerated. NK cells were enriched using the NK cell isolation kit II (Miltenyi, UK) according to the manufacturers protocol. Isolated NK cells were culture at 1 × 10^6/ml in T cells media with high dose IL-2 (1000U/ml) human IL-2 (Peprotech, UK) for 4 days. After four days cells were washed and used in an LDH release assay (Promega) following the manufacturers protocol. YAC-1 cells (a kind gift from Professor Gillian Griffiths, University of Cambridge, Cambridge, UK) were used as target cells in the assay. YAC-1s were maintained in RPMI, 10% fetal calf serum and 10U/ml Penicillin Streptomycin. 10^4 targets were used at a starting effector:target ratio of 5:1.

Infection studies

Infection of mice was carried out as part of the pathogen-screening pipeline in the mouse genetics project (5). Age and sex matched *Nbeal2*$^{+/+}$ or *Nbeal2*$^{-/-}$ mice were infected *i.v.* with 0.2 ml *Salmonella enterica* serovar Typhimurium M525 (phoN::tetC) containing 5 x 10^5 CFU of bacteria in sterile PBS. Mice were monitored as outlined in the mouse genetics project and results were made available through the International Mouse Phenotyping Consortium (http://www.mousephenotype.org/). For infection with virulent *Salmonella enterica* serovar Typhimurium SL1344, mice were orally infected with 10^9 CFU of bacteria. For infection with *Staphylococcus aureus*, mice were infected i.v. with 4 x 10^7 CFU of bacteria as established previously (6). MCMV was cultured as described before (3), and 3 x 10^4 PFU of virus was used to infect mice *i.p.* For neutrophil depletion studies, mice were administered with 300µg InVivoPlus anti-mouse Ly6G (Clone: 1A8) or InVivoPlus Rat IgG2a Isotype control (both BioXCell, USA) *i.p.* on days -1 and +1 post infection. Mice were sacrificed on day 3 post-infection. NK cells were depleted as described previously (7). In brief, mice were administered

with 250μg InVivoMAb anti-mouse NK1.1 (Clone: PK136) or InVivoMAb Mouse IgG2a Isotype control (Clone: C1.18.4) *i.p* on days -2, 0 and +2 post-infection. Mice were sacrificed as before on day 4 post-infection. For all infections mice were monitored daily for changes in weight and mice were culled if they lost more than 20% of their body weight. Otherwise, mice were sacrificed at time points indicated in the main text and spleen, liver, kidneys, blood, caecal contents were harvested/collected. Bacterial CFUs for each tissue was quantified using serial dilution and plating onto agar plates. MCMV titres were calculated as described previously (7).

Histology

Mouse kidneys from *S. aureus* infected isotype control treated *Nbeal2*$^{+/+}$ and *Nbeal2*$^{-/-}$ (described in Infection Studies) were removed and immediately fixed in 10% formalin when the mice were culled. Kidney's were processed, sectioned (2μm thickness), stained (haematoxylin and eosin) and analysed as part of the core histology service at the Metabolic Research Laboratories (Addenbrooke's, Cambridge).

Statistics

Statistical analysis was carried out using GraphPad Prism (Prism version 5.00 for Mac OS X, GraphPad Software, La Jolla California USA, www.graphpad.com). Mann-Whitney U unpaired t-tests (2-tailed), Kruskal-Wallis one-way ANOVAs, 2-way ANOVAs or log-rank tests were used as indicated in the figure legends. Significance threshold of $P = 0.05$ was used throughout.

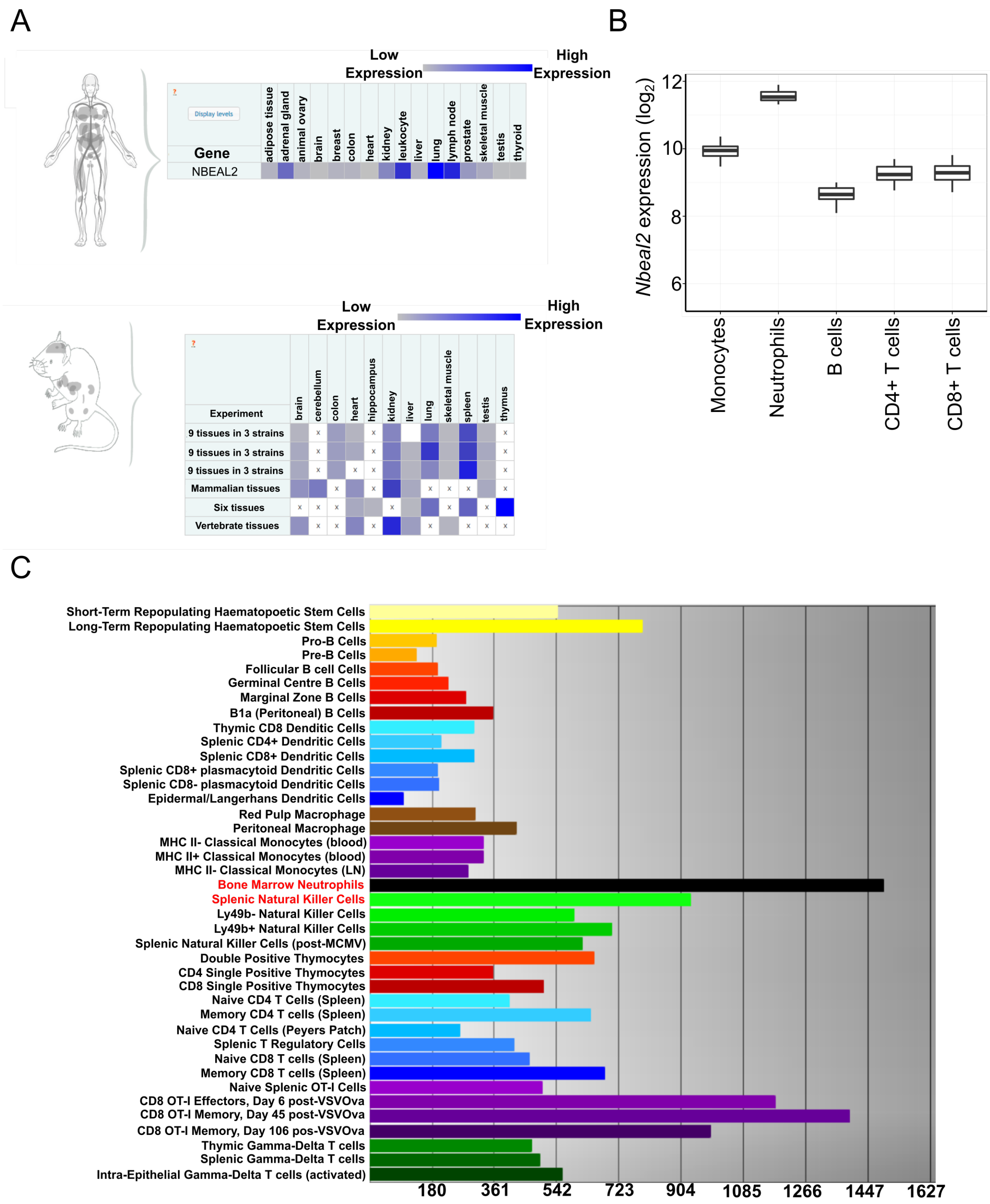

Supplemental Figure 1.

***Nbeal2* is highly expressed in the immune system of mice and humans**. (**A**) The gene expression atlas (https://www.ebi.ac.uk/gxa/home) was used to analyse expression of *Nbeal2* mRNA in multiple tissues of both human (top) and mouse (bottom). (**B**) Human leukocyte subsets were isolated from peripheral blood of healthy volunteers, mRNA was extracted and the whole transcriptome was analysed using microarrays. Box plots show the expression ($\log_2$) of Nbeal2 in each of the different leukocyte subsets. (**C**) *Nbeal2* mRNA levels were assessed within different murine immune cells using the ImmGen database (https://www.immgen.org/). Expression was measured by microarray from a standardised flow sorting and microarray analysis pipeline (2). Expression is presented as a normalised linear value. Expression in natural killer cells and bone marrow neutrophils is highlighted in red.

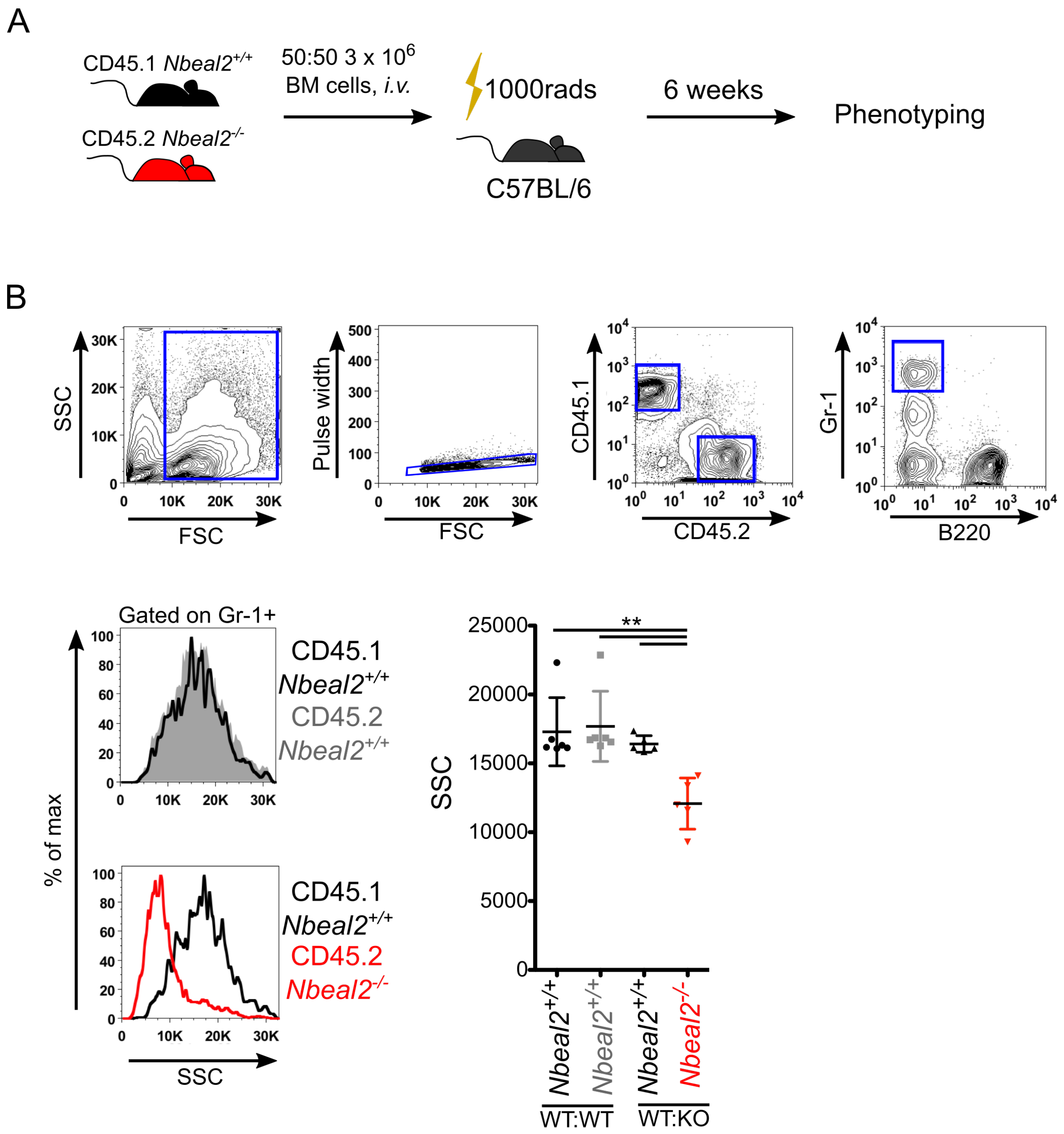

Supplemental Figure 2.

Side-scatter abnormality in *Nbeal2*$^{-/-}$ neutrophils is cell intrinsic. (**A**) Schematic showing how mixed bone marrow chimeras were setup. A 50:50 mixture of congenically (CD45.1 and CD45.2) marked bone marrow from *Nbeal2*$^{+/+}$ and *Nbeal2*$^{-/-}$ mice was injected *i.v.* into lethally irradiated CD57BL/6 mice. After six weeks of reconstitution, mice were sacrificed and immunophenotyped. (**B**) Gating strategy of whole blood analysis in the mixed bone marrow chimeras. White blood cells were gated on using forward scatter (FSC) and side scatter (SSC) profiles, and singlets were excluded with pulse width discrimination. Geometric mean of the SSC of Gr-1^{hi} B220- cells in the peripheral blood was measured and analysed in GraphPad. Each dot represents an individual mouse. Data shows mean and s.d. ** $P < 0.01$, 2-tailed Mann Whitney U test.

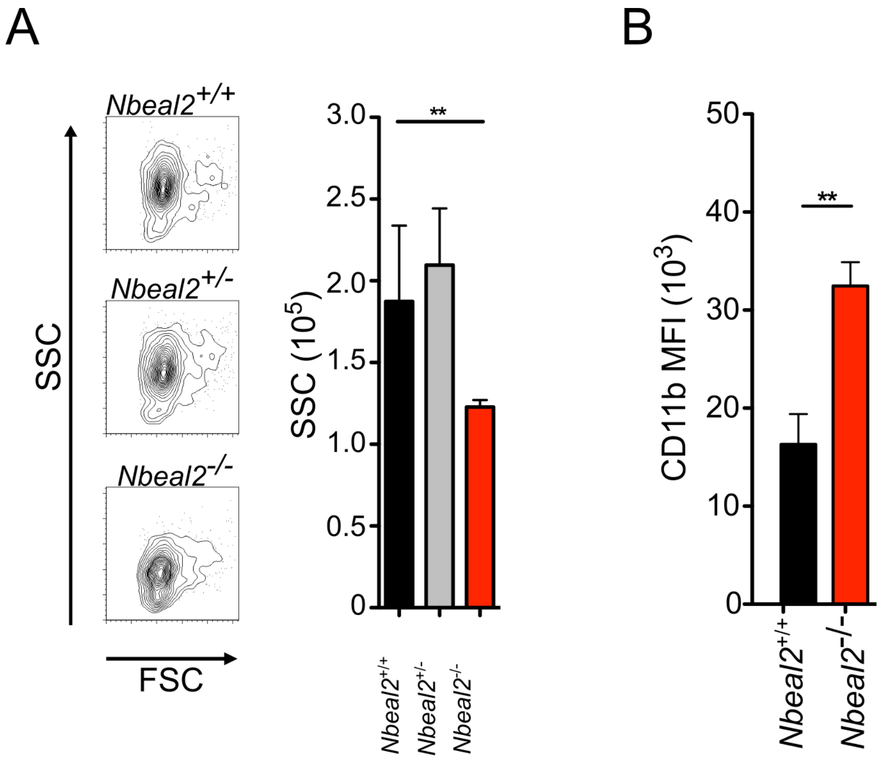

Supplemental Figure 3.

Altered phenotype of *Nbeal2*$^{-/-}$ granulocytes. (**A**) Reduced side-scatter (SSC) in SiglecF+ CD11b+ splenic eosinophils. Representative FACS plots on the left showing SSC versus forward scatter (FSC) together with the geometric mean of SSC quantified on the right (n = 6-7). (**B**) Geometric mean fluorescence intensity of CD11b on bone marrow neutrophils. Data are presented as mean and error bars showing ± s.d. Data are representative of 2-3 independent experiments. ** P < 0.01, Kruskal-Wallis one-way ANOVA (A) 2-tailed Mann-Whitney U test (B).

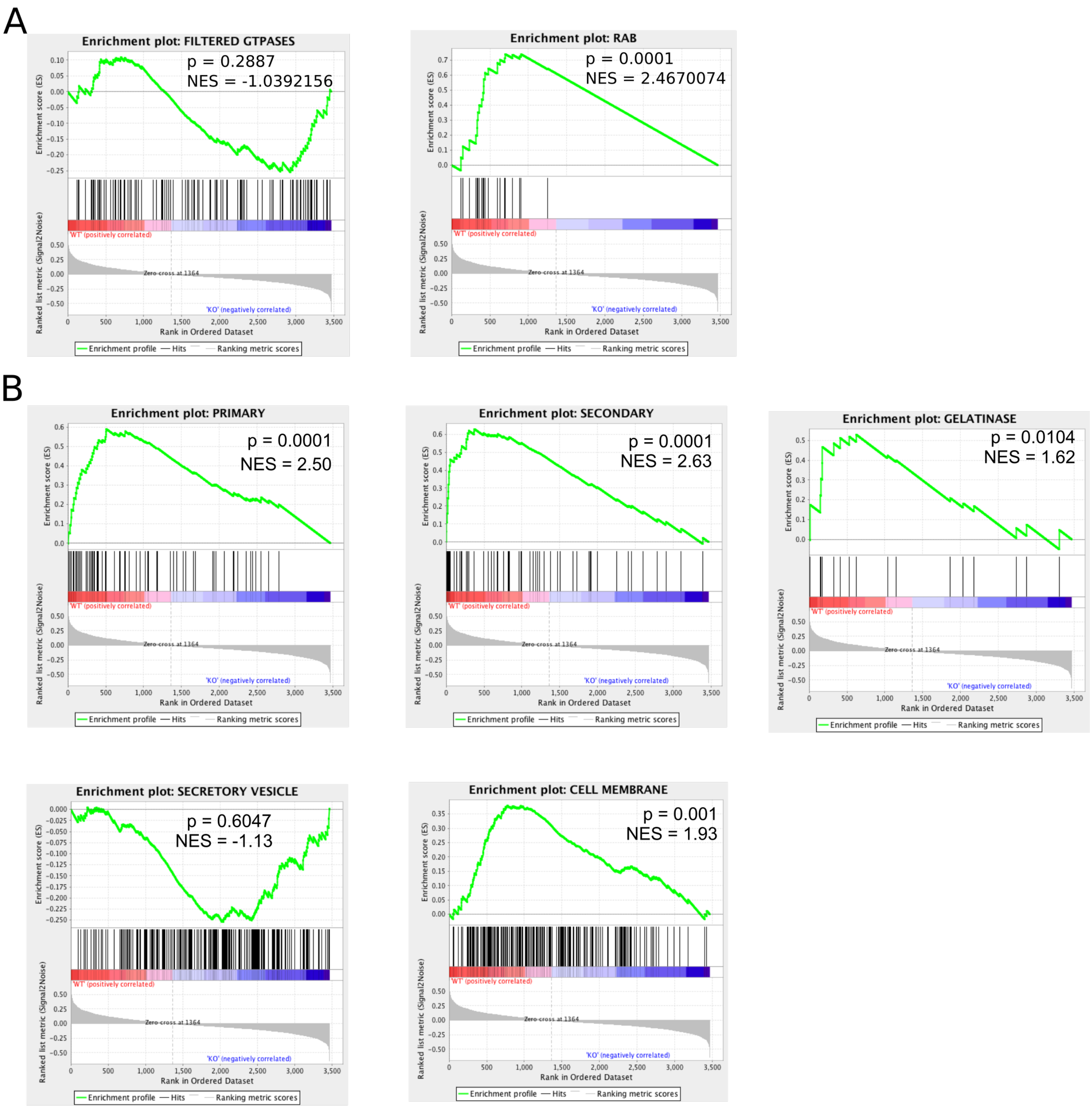

Supplemental Figure 4.

Protein Set Enrichment Analysis (PSEA) of proteins in *Nbeal2*$^{+/+}$ and *Nbeal2*$^{-/-}$ neutrophils. (A) The filtered and normalised proteome expression set for control and knockout neutrophils was analysed using the GSEA software as described in the methods. The approximately 3500 proteins expressed in both *Nbeal2*$^{+/+}$ and *Nbeal2*$^{-/-}$ neutrophils were analysed for enrichment of either total GTPases expressed in neutrophils (left) or Rab family members (right). Displayed is the FDR corrected p-value as well as the normalised enrichment score (NES). (B) Similar analysis using proteins identified in primary, secondary, and gelatinase granules, as well as secretory vesicles and on the membrane fraction of human neutrophils (8).

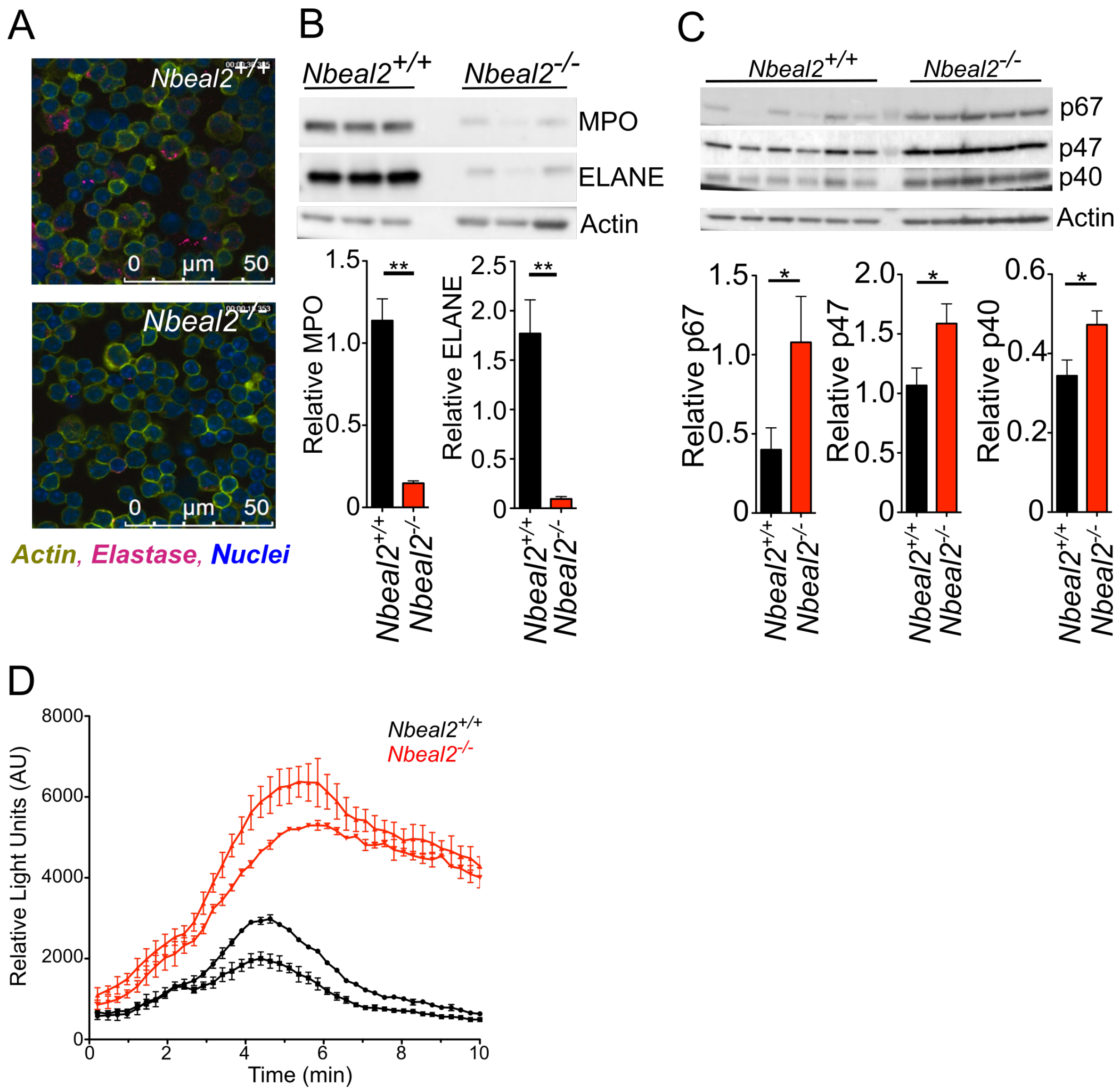

Supplemental Figure 5

***In vitro* characterisation of *Nbeal2*$^{-/-}$ neutrophils.** (**A**) Immunofluorescence of elastase in isolated BM neutrophils in either *Nbeal2*$^{+/+}$ (top) or *Nbeal2*$^{-/-}$ (bottom) mice. Representative image of each genotype is shown. F-actin was stained using rhodamine-phalloidin (yellow), an anti-mouse elastase (pink) and nuclei were stained using DAPI (blue). (**B**) Representative western blot images of BM neutrophils lysed in loading buffer and blotted for expression of myeloperoxidase (MPO) and elastase (ELANE) or (**C**) the cytosolic NADPH oxidase components p67 (*Ncf2*), p47 (*Ncf1*) and p40 (*Ncf4*). β-Actin was used as a loading control. Shown below are the densitometries calculated using ImageLab software and expression is normalised to β-Actin (n = 3-6, pooled data from two independent experiments). (**D**) BM neutrophils from *Nbeal2*$^{+/+}$ and *Nbeal2*$^{-/-}$ were stimulated with PMA and reactive oxygen species were measured using the chemiluminescent substrate lucigenin. Data are representative of 2 mice with technical duplicates for each. Data are presented as mean and s.d (B and C) or s.e.m. (D), * P < 0.05, 2-tailed Mann-Whitney U test.

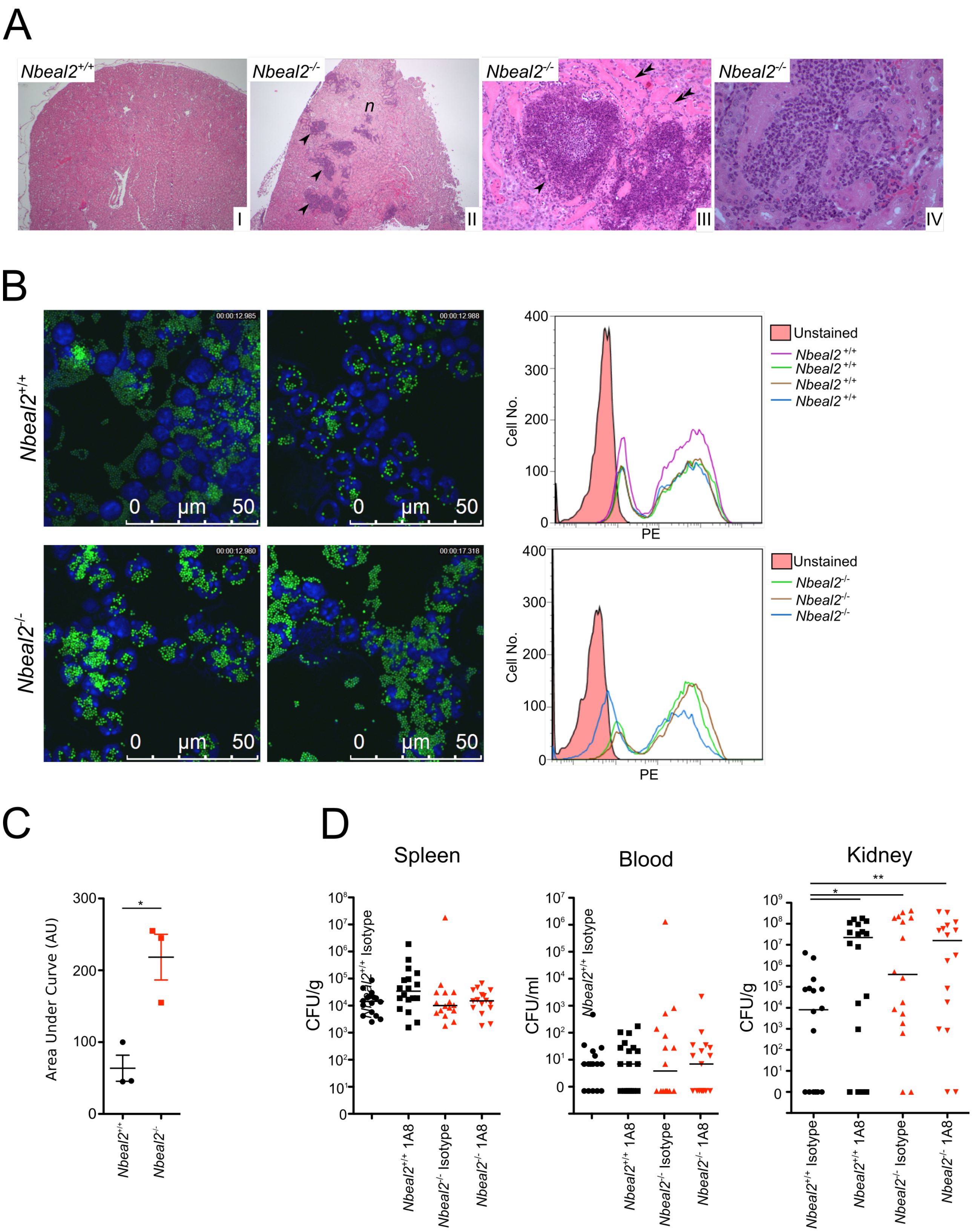

Supplemental Figure 6

Response of *Nbeal2*$^{-/-}$ mice to *S. Aureus*. (**A**) Kidney histology (haematoxylin and eosin staining) of *S. aureus* infected *Nbeal2*$^{+/+}$ and *Nbeal2*$^{-/-}$ mice at day 3 post-infection. Panel I shows *Nbeal2*$^{+/+}$ kidney showing no overt pathological abnormalities (X40). Panels II-IV shows *Nbeal2*$^{-/-}$ kidney sections with various signs of pathology. II shows tissue necrosis (n) as well as several bacterial microabscesses

(arrowheads) (X40). III shows focal abscess formation (arrowhead) and surrounding area of acute tubular necrosis (double arrowheads) (X200). IV shows an increased magnification of a bacteria-rich inflammatory lesion with monocytic and neutrophilic infiltrate (X400). (**B**) *In vitro* phagocytosis of fluorescently labelled S. aureus by MACS purified bone marrow neutrophils from either C57BL/6 control (top panels) or *Nbeal2*$^{-/-}$ mice (bottom panels). Representative fluorescence microscopy images are shown together with FACS quantification of phagocytosed bacteria. (n = 3-4). (**C**) Bone marrow neutrophils were stimulated with serum-opsonised S. aureus at a ratio of 20:1 (bacteria:cells) and reactive oxygen species were measured using the chemiluminescent substrate lucigenin. Graph shows the area under the curve. (n = 3) (**D**) *Nbeal2*$^{+/+}$ and *Nbeal2*$^{-/-}$ mice were treated with an isotype control antibody or an anti-Ly6G antibody (1A8) that specifically depletes neutrophils (9), on days -1, and +1 post-infection with 4×10^7 of *S. aureus* sh1000. Mice were sacrificed on day 3 post-infection and bacterial counts were determined from blood, spleen, and kidney (n = 10-13). Data shows mean and s.e.m. (**C**) or median (**D**). Data in (**D**) are representative of 2 pooled independent experiments. * $P < 0.05$, 2-tailed student's t-test (**C**) or 2-tailed Mann-Whitney U test (**D**).

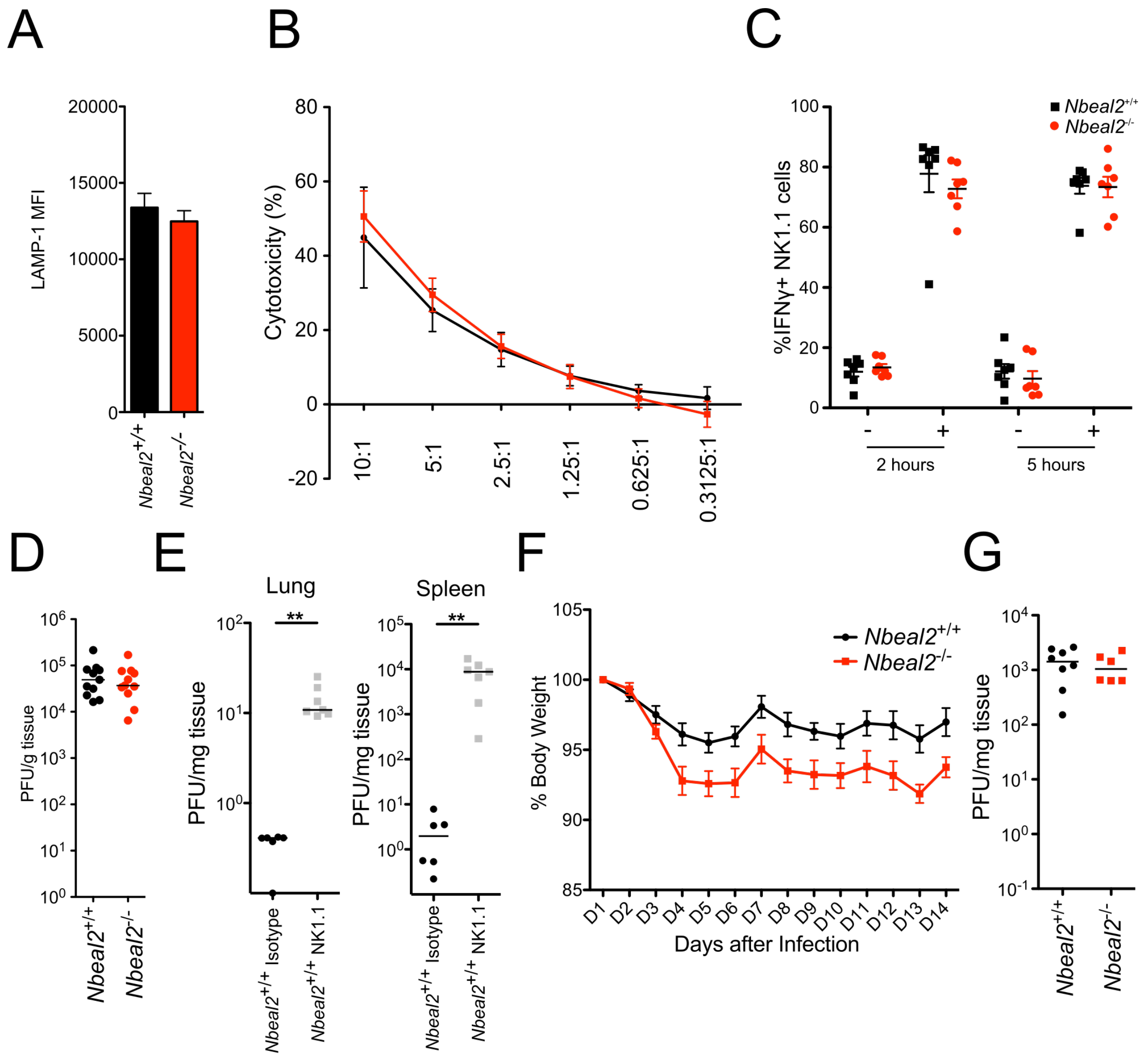

Supplemental Figure 7

NK functionality, CD8 cytotoxicity and longer term in vivo mCMV experiments

(A) Splenic NK cells (CD3-, B220-, NK1.1+, NKp46+) were stained intracellularly for LAMP-1 as described in Materials and Methods. The geometric mean fluorescence is shown for both *Nbeal2*$^{+/+}$ and *Nbeal2*$^{-/-}$ NK cells (n=5-7). (**B**) Effect of NK depletion on mCMV infection. Mice were treated with an isotype control antibody or an anti-NK1.1 antibody on days -2, 0 and +2 post-infection, and infected as before. On day 4 mice were culled and viral plaque assays were used to determine PFUs in the lung and spleen of infected mice. n = 6. (**C**) Splenocytes from *Nbeal2*$^{+/+}$ or *Nbeal2*$^{-/-}$ mice were used in a cytotoxicity assay that measures lactose dehydrogenase release by the P815 target cell line, as described in the Methods. Displayed is the % cytotoxicity at different cell:target ratios. n = 3. (**D**) FACS plots showing the percentage of IFN-γ positive NK cells (gated on NK1.1+) from splenocytes of mCMV infected mice at day 4 post-infection. Cells were stimulated with PMA/ionomycin for the times indicated, in the presence of brefeldin A. n = 7. (**E**) Pooled plaque-forming units (PFUs) of virus at day 4 post-infection in the livers of mCMV infected *Nbeal2*$^{+/+}$ and *Nbeal2*$^{-/-}$ mice. n = 10-11. Mice were infected as before but experiment was run for 14 days post-infection. Shown are the changes in weight (**F**) and the PFUs of mouse salivary glands (**G**) at day 14. n = 6-8. Data shows mean and s.e.m. (A, C-D, and F) or median (B, E, G). Data representative of two independent experiments (C and F). * $P < 0.05$, 2-tailed Mann-Whitney U test (**B**).

Supplemental Table 1. Total proteome identified in *Nbeal2+/+* and *Nbeal2-/-* neutrophils analysed by limma differential protein analysis (*Nbeal2+/+* / *Nbeal2-/-*)

protID	logFC	AveExpr	t	P.Value	adj.P.Val	B
Grlf1	-6.68	26.3	-13.7	0.00000	0.00038	5.75
Nap1l4	-5.55	26.0	-10.8	0.00001	0.00057	4.24
Naca	-5.51	27.3	-12.0	0.00000	0.00047	4.84
Rps11	-4.89	27.0	-5.8	0.00060	0.00507	0.09
Aak1	-4.72	26.2	-10.6	0.00001	0.00060	4.15
Atp6v1g1	-4.72	26.4	-12.4	0.00000	0.00045	5.12
Alyref	-4.69	26.4	-7.5	0.00011	0.00194	1.81
Rpl9	-4.67	26.3	-11.0	0.00001	0.00057	4.39
Ppm1f	-4.56	25.1	-11.0	0.00001	0.00057	4.35
St13	-4.50	25.6	-8.7	0.00004	0.00113	2.85
Rpl18	-4.49	26.3	-4.3	0.00346	0.01549	-1.74
Bola2	-4.47	24.9	-9.2	0.00003	0.00099	3.18
Rpl22	-4.41	27.1	-4.0	0.00511	0.02016	-2.15
Dynlrb1	-4.39	25.9	-5.0	0.00146	0.00870	-0.84
Asprv1	-4.27	27.4	-7.7	0.00009	0.00180	1.98
Golga3	-4.25	25.7	-8.7	0.00004	0.00115	2.81
Dync1li1	-4.25	27.1	-8.9	0.00004	0.00112	2.89
Map4	-4.21	27.3	-4.8	0.00172	0.00980	-1.01
Snrpa1	-4.14	26.0	-8.5	0.00005	0.00120	2.68
G3bp1	-4.04	26.2	-9.9	0.00002	0.00076	3.67
Qki	-4.03	25.3	-6.8	0.00020	0.00268	1.21
Abhd5	-3.99	25.5	-7.2	0.00015	0.00230	1.52
Dnajc9	-3.94	25.2	-9.5	0.00002	0.00087	3.40
Kpna4	-3.93	26.1	-5.7	0.00062	0.00522	0.05
Nup35	-3.93	26.1	-6.7	0.00023	0.00282	1.08
Ccdc22	-3.89	25.3	-5.6	0.00070	0.00564	-0.08
Pabpc1	-3.88	30.5	-8.7	0.00004	0.00116	2.77
Mob1a	-3.87	28.0	-3.2	0.01448	0.04180	-3.24
Atp6v1f	-3.85	25.4	-6.3	0.00034	0.00356	0.68
Rpl38	-3.80	25.1	-7.7	0.00009	0.00180	1.98
Gatad2b	-3.78	25.0	-8.6	0.00004	0.00115	2.77
Crot	-3.78	25.6	-7.3	0.00013	0.00211	1.65
Arhgap1	-3.77	28.6	-5.1	0.00126	0.00795	-0.69
Hdgfrp2	-3.75	24.6	-4.8	0.00180	0.01007	-1.06
Hist1h1c	-3.74	28.6	-6.6	0.00027	0.00310	0.93
Lsp1	-3.71	25.1	-7.9	0.00008	0.00161	2.18
Irf2bp2	-3.70	25.6	-8.0	0.00007	0.00152	2.30
Rpl10	-3.69	25.3	-8.0	0.00007	0.00153	2.27
Rps25	-3.66	25.7	-8.6	0.00004	0.00115	2.78
Pafah1b2	-3.66	24.9	-7.0	0.00016	0.00243	1.41
Rps10	-3.66	26.3	-4.6	0.00239	0.01210	-1.36
Eif1	-3.61	27.0	-4.9	0.00153	0.00900	-0.89
Rps17	-3.61	26.5	-6.4	0.00030	0.00342	0.79
Gcc2	-3.59	27.8	-4.4	0.00300	0.01423	-1.60
Sumo2	-3.58	27.6	-3.1	0.01569	0.04431	-3.32
Nasp	-3.58	25.5	-7.0	0.00017	0.00246	1.37
Lcp2	-3.55	27.2	-9.3	0.00002	0.00092	3.29
Rpl27	-3.54	25.2	-8.1	0.00006	0.00143	2.38
Rplp2	-3.52	27.2	-4.7	0.00205	0.01101	-1.20
Fbp1	-3.51	26.4	-6.0	0.00047	0.00434	0.33
Usp25	-3.50	26.2	-8.6	0.00004	0.00118	2.72
Nck1	-3.48	26.3	-6.4	0.00032	0.00349	0.74
Serbp1	-3.48	26.1	-5.1	0.00130	0.00808	-0.72
Pnp2	-3.47	24.7	-7.2	0.00013	0.00215	1.61
Strn4	-3.47	25.2	-3.1	0.01769	0.04844	-3.44
Fam101b	-3.45	27.1	-7.2	0.00015	0.00235	1.49
Rpl5	-3.45	26.9	-5.3	0.00103	0.00697	-0.47
Eif3b	-3.45	28.2	-11.6	0.00001	0.00048	4.71
Eif2s3y	-3.44	25.1	-7.8	0.00008	0.00172	2.08
Csnk2a2	-3.44	24.9	-4.6	0.00232	0.01190	-1.33

Dera	-3.43	25.3	-7.5	0.00011	0.00190	1.85
Wipi1	-3.39	24.9	-4.8	0.00174	0.00983	-1.02
Hnrnpa0	-3.38	26.4	-3.9	0.00546	0.02126	-2.22
Prrc2c	-3.38	25.8	-6.1	0.00040	0.00390	0.48
Tnks1bp1	-3.36	25.7	-4.1	0.00446	0.01840	-2.01
Rap1gds1	-3.36	26.6	-8.8	0.00004	0.00112	2.88
Ehbp1l1	-3.36	26.3	-8.0	0.00007	0.00152	2.29
Dak	-3.35	24.6	-7.0	0.00016	0.00243	1.42
Rpl30	-3.33	25.9	-3.5	0.00885	0.02952	-2.73
Ranbp9	-3.33	25.3	-3.4	0.01035	0.03311	-2.89
Gm2056	-3.30	24.8	-6.4	0.00031	0.00344	0.77
Ddx1	-3.29	27.0	-10.4	0.00001	0.00065	4.02
Sh3kbp1	-3.29	28.2	-4.8	0.00185	0.01019	-1.09
Hmgb1	-3.28	29.3	-6.9	0.00020	0.00268	1.21
Cope	-3.28	26.8	-4.7	0.00201	0.01085	-1.18
Rps18	-3.28	29.6	-14.2	0.00000	0.00038	5.94
Hist2h2bb	-3.27	25.4	-6.0	0.00047	0.00431	0.33
Cntrl	-3.26	27.8	-7.6	0.00010	0.00187	1.91
Rps19	-3.26	27.8	-3.6	0.00782	0.02697	-2.60
Tpm3	-3.24	25.3	-4.2	0.00383	0.01665	-1.85
Crkl	-3.24	26.3	-4.6	0.00221	0.01152	-1.27
Gmppb	-3.23	26.3	-3.6	0.00875	0.02928	-2.71
Yy1	-3.22	24.4	-8.0	0.00007	0.00155	2.24
Trappc4	-3.22	25.2	-5.3	0.00092	0.00655	-0.37
Epg5	-3.20	25.1	-5.4	0.00085	0.00620	-0.29
Pak1	-3.20	24.3	-6.7	0.00022	0.00275	1.12
Map2k2	-3.16	25.3	-4.7	0.00196	0.01063	-1.15
Atp6v1d	-3.16	25.3	-6.2	0.00036	0.00369	0.61
Naa50	-3.15	24.6	-7.3	0.00013	0.00209	1.67
Nup43	-3.14	24.8	-6.4	0.00031	0.00346	0.76
Ccdc88c	-3.14	25.8	-4.0	0.00459	0.01866	-2.04
Lars	-3.14	26.3	-5.7	0.00065	0.00539	0.00
Prps2	-3.13	26.3	-3.1	0.01712	0.04724	-3.41
Anapc1	-3.12	25.3	-5.8	0.00054	0.00488	0.18
Ints1	-3.12	26.5	-4.9	0.00159	0.00923	-0.93
Rprd2	-3.10	26.7	-5.6	0.00074	0.00581	-0.13
Kpna3	-3.10	25.1	-6.3	0.00032	0.00349	0.73
Gabarap	-3.09	25.3	-4.6	0.00231	0.01189	-1.32
Psmd11	-3.09	28.3	-6.0	0.00047	0.00431	0.34
Arid4a	-3.08	25.4	-6.8	0.00020	0.00268	1.19
Galk1	-3.08	26.4	-4.2	0.00374	0.01638	-1.83
Aimp1	-3.07	26.8	-3.5	0.00942	0.03088	-2.79
Ppil1	-3.07	26.2	-5.0	0.00137	0.00836	-0.78
Trip11	-3.06	27.9	-10.1	0.00001	0.00072	3.81
Ddx39	-3.05	25.3	-3.8	0.00638	0.02348	-2.38
Sptbn1	-3.04	25.7	-5.5	0.00079	0.00601	-0.22
Sec16a	-3.03	28.0	-9.5	0.00002	0.00087	3.39
Trip12	-3.02	26.8	-5.1	0.00128	0.00806	-0.71
Sptan1	-3.02	25.7	-4.7	0.00204	0.01096	-1.19
Nedd9	-3.02	26.5	-7.2	0.00015	0.00228	1.53
Nin	-3.01	27.5	-5.3	0.00105	0.00706	-0.49
Zc3h18	-3.00	26.0	-6.2	0.00036	0.00370	0.60
Gtf2f2	-2.98	24.5	-4.9	0.00154	0.00902	-0.90
Arid1b	-2.97	24.7	-7.2	0.00014	0.00218	1.59
Synrg	-2.97	25.6	-5.5	0.00075	0.00582	-0.16
Pik3r4	-2.96	24.3	-6.4	0.00029	0.00324	0.84
Btf3	-2.95	25.7	-3.9	0.00581	0.02218	-2.29
Arpc5l	-2.95	25.1	-5.5	0.00078	0.00599	-0.20
Dck	-2.94	24.5	-5.6	0.00071	0.00564	-0.09
Ddx42	-2.94	24.8	-6.1	0.00040	0.00390	0.48
Specc1	-2.94	26.8	-4.0	0.00491	0.01956	-2.11
6430548M08Rik	-2.94	27.1	-5.3	0.00096	0.00668	-0.40
Lsm3	-2.93	25.7	-7.4	0.00012	0.00206	1.71
Lypla1	-2.93	26.7	-3.6	0.00841	0.02861	-2.67

Pdcd5	-2.92	25.1	-4.9	0.00151	0.00893	-0.89
Cops8	-2.92	25.4	-5.9	0.00052	0.00473	0.22
Nans	-2.92	27.4	-7.0	0.00018	0.00249	1.35
Ikbkg	-2.92	24.8	-4.1	0.00410	0.01744	-1.92
Pabpn1	-2.91	24.9	-5.7	0.00059	0.00505	0.09
Sf3b4	-2.91	24.9	-5.6	0.00070	0.00563	-0.09
Rbm22	-2.91	24.4	-3.6	0.00849	0.02884	-2.68
Rps3	-2.90	30.6	-15.8	0.00000	0.00031	6.57
Bid	-2.89	26.7	-4.2	0.00375	0.01641	-1.83
Ran	-2.87	31.3	-11.9	0.00000	0.00047	4.84
Sp1	-2.85	24.5	-5.6	0.00067	0.00549	-0.05
Fkbp8	-2.85	24.0	-4.1	0.00427	0.01791	-1.97
Mov10	-2.84	25.3	-4.7	0.00185	0.01019	-1.10
Akap9	-2.83	25.2	-3.9	0.00564	0.02165	-2.26
Golga4	-2.83	27.3	-5.9	0.00054	0.00488	0.20
Ppid	-2.82	27.8	-10.1	0.00001	0.00072	3.81
Babam1	-2.80	24.9	-5.5	0.00078	0.00599	-0.20
Fcho2	-2.79	25.3	-5.2	0.00112	0.00742	-0.58
Eftud1	-2.78	24.1	-5.8	0.00058	0.00505	0.11
Nupl1	-2.78	25.9	-6.2	0.00035	0.00365	0.63
Rps16	-2.78	27.2	-4.2	0.00364	0.01613	-1.80
Reps1	-2.76	25.7	-5.0	0.00129	0.00808	-0.73
Rpl6	-2.76	29.0	-10.2	0.00001	0.00072	3.89
Ubtf	-2.76	25.4	-5.6	0.00068	0.00550	-0.05
Nap1l1	-2.75	26.8	-3.5	0.00959	0.03129	-2.81
Prrc2a	-2.74	25.5	-3.7	0.00748	0.02610	-2.55
Cpsf3	-2.74	26.0	-4.3	0.00341	0.01540	-1.73
Ndrg1	-2.74	25.2	-5.1	0.00122	0.00782	-0.67
Lamtor3	-2.74	26.5	-3.5	0.00930	0.03060	-2.78
Arhgap9	-2.72	27.2	-3.1	0.01645	0.04569	-3.37
Carhsp1	-2.72	25.3	-3.6	0.00796	0.02736	-2.62
Dpp9	-2.72	25.5	-3.2	0.01489	0.04258	-3.26
Rpa3	-2.71	24.8	-4.0	0.00462	0.01872	-2.05
Dnaja1	-2.70	26.2	-3.8	0.00596	0.02247	-2.31
Actb	-2.70	33.2	-6.1	0.00042	0.00402	0.46
Ncf4	-2.69	30.7	-6.3	0.00035	0.00365	0.64
Pak2	-2.69	29.9	-9.4	0.00003	0.00095	3.25
Vcpip1	-2.68	24.5	-5.8	0.00058	0.00505	0.10
Tiprl	-2.68	26.0	-4.0	0.00486	0.01942	-2.10
Rassf5	-2.68	25.0	-6.0	0.00047	0.00431	0.33
Inpp1	-2.67	26.9	-3.2	0.01356	0.03997	-3.17
Eef1e1	-2.67	25.0	-5.4	0.00085	0.00621	-0.29
Hspa14	-2.66	25.9	-5.4	0.00082	0.00611	-0.25
Fyb	-2.66	28.1	-6.4	0.00031	0.00347	0.76
U2af1	-2.65	25.8	-4.9	0.00146	0.00871	-0.85
Col4a3bp	-2.65	25.0	-3.8	0.00590	0.02237	-2.30
Rnf114	-2.64	25.0	-3.9	0.00550	0.02131	-2.23
Rtcb	-2.63	26.8	-7.5	0.00011	0.00194	1.80
Qil1	-2.63	24.1	-6.7	0.00022	0.00281	1.09
Sh3bp1	-2.63	26.8	-6.5	0.00028	0.00322	0.85
Map2k1	-2.63	27.7	-4.6	0.00218	0.01147	-1.26
Scyl2	-2.62	25.3	-3.1	0.01722	0.04747	-3.41
Strn	-2.62	27.7	-7.5	0.00010	0.00189	1.87
Rps4x	-2.62	28.2	-10.2	0.00001	0.00072	3.87
Srsf9	-2.61	25.3	-3.8	0.00590	0.02237	-2.30
Ranbp1	-2.61	27.9	-6.2	0.00038	0.00378	0.55
Hspa4l	-2.61	26.7	-3.6	0.00776	0.02682	-2.59
Cep128	-2.61	26.6	-5.3	0.00098	0.00678	-0.44
Vps13a	-2.61	26.0	-3.1	0.01672	0.04637	-3.38
Map4k2	-2.61	25.1	-3.7	0.00697	0.02498	-2.48
Hectd3	-2.60	25.6	-3.7	0.00708	0.02526	-2.49
Cep250	-2.59	26.0	-4.5	0.00245	0.01222	-1.39
Thada	-2.59	24.2	-3.3	0.01274	0.03818	-3.10
Map2k4	-2.59	26.0	-5.1	0.00115	0.00753	-0.60

Usp47	-2.58	26.6	-4.6	0.00226	0.01169	-1.30
Ywhab	-2.57	31.7	-13.4	0.00000	0.00038	5.63
Psmg1	-2.57	25.6	-5.4	0.00083	0.00613	-0.26
Arid1b	-2.57	27.4	-7.2	0.00013	0.00215	1.61
Tjp1	-2.57	25.4	-4.6	0.00218	0.01147	-1.27
Ctdp1	-2.57	24.3	-6.8	0.00020	0.00268	1.20
Cbx5	-2.56	26.6	-4.3	0.00335	0.01528	-1.71
Lcmt1	-2.56	24.3	-5.5	0.00079	0.00599	-0.21
Pdcl3	-2.55	26.1	-3.2	0.01404	0.04103	-3.20
Rbm14	-2.55	28.9	-10.9	0.00001	0.00057	4.30
Mif	-2.55	26.6	-4.4	0.00305	0.01445	-1.61
Rpl12	-2.55	29.3	-11.1	0.00001	0.00057	4.43
Nup133	-2.54	27.5	-3.3	0.01228	0.03725	-3.07
Abcf1	-2.54	25.0	-3.8	0.00632	0.02345	-2.37
Psmd14	-2.53	27.5	-4.7	0.00210	0.01121	-1.22
Ncoa3	-2.53	25.1	-4.3	0.00338	0.01534	-1.73
Psmd7	-2.52	27.1	-5.3	0.00101	0.00690	-0.46
Fam63b	-2.52	24.0	-6.8	0.00021	0.00269	1.16
Stip1	-2.52	30.1	-10.8	0.00001	0.00057	4.25
Usp24	-2.52	28.0	-5.5	0.00078	0.00599	-0.19
Ubxn6	-2.51	24.5	-5.1	0.00116	0.00755	-0.61
Ptpn23	-2.51	25.6	-5.0	0.00129	0.00808	-0.72
Nudt5	-2.50	25.1	-4.6	0.00225	0.01165	-1.30
Arfgef2	-2.50	27.5	-6.2	0.00038	0.00378	0.54
Exoc1	-2.50	25.0	-4.0	0.00451	0.01845	-2.03
Ccar1	-2.49	28.2	-6.3	0.00033	0.00355	0.70
1110037F02Rik	-2.49	25.1	-4.5	0.00249	0.01234	-1.41
Wdr26	-2.49	25.7	-3.2	0.01439	0.04170	-3.23
Msh2	-2.49	25.2	-5.0	0.00136	0.00831	-0.78
Col6a3	-2.47	27.1	-3.4	0.01054	0.03342	-2.91
Bcas2	-2.47	25.8	-5.1	0.00122	0.00782	-0.67
RBM8	-2.47	25.8	-5.8	0.00058	0.00505	0.12
Gars	-2.45	28.3	-7.6	0.00011	0.00189	1.87
Arhgdia	-2.45	31.7	-7.1	0.00016	0.00243	1.43
Baz1a	-2.44	26.4	-4.1	0.00407	0.01733	-1.91
Adk	-2.44	25.4	-4.6	0.00217	0.01144	-1.27
Med23	-2.44	24.7	-4.5	0.00256	0.01262	-1.44
Fip1l1	-2.44	26.3	-5.6	0.00069	0.00560	-0.08
Acot8	-2.44	24.3	-6.1	0.00043	0.00406	0.43
Asap1	-2.44	26.3	-5.7	0.00060	0.00507	0.08
Ylpm1	-2.43	27.4	-6.8	0.00020	0.00268	1.20
Ssh2	-2.43	24.7	-5.3	0.00100	0.00685	-0.46
Arap3	-2.43	25.3	-4.0	0.00442	0.01832	-2.01
Ptgr2	-2.43	24.4	-5.8	0.00058	0.00505	0.10
Rpl28	-2.42	27.4	-9.0	0.00003	0.00106	3.04
Otub1	-2.42	28.4	-6.6	0.00026	0.00310	0.94
Ppil4	-2.41	24.6	-5.2	0.00112	0.00743	-0.58
Dctn3	-2.41	25.1	-3.4	0.01022	0.03283	-2.87
Sri	-2.40	30.6	-6.4	0.00031	0.00342	0.78
Ak2	-2.40	28.8	-8.8	0.00004	0.00112	2.91
Tcof1	-2.40	24.3	-5.3	0.00101	0.00690	-0.47
Hist1h2bj	-2.39	34.6	-5.0	0.00136	0.00833	-0.77
Swap70	-2.39	25.9	-4.0	0.00445	0.01839	-2.02
Kntc1	-2.38	27.1	-6.4	0.00031	0.00347	0.75
Pik3c3	-2.38	24.4	-3.5	0.00911	0.03015	-2.76
Psmd9	-2.38	26.7	-6.5	0.00028	0.00320	0.86
Nup88	-2.38	24.9	-4.3	0.00338	0.01534	-1.73
Sec62	-2.38	25.3	-4.5	0.00243	0.01220	-1.39
Mtmr3	-2.37	28.6	-9.8	0.00002	0.00076	3.64
Acaca	-2.37	27.9	-7.7	0.00009	0.00177	2.02
Gm20390	-2.37	31.2	-8.2	0.00006	0.00142	2.40
Psmc6	-2.37	28.5	-4.6	0.00235	0.01195	-1.34
Tpt1	-2.37	28.7	-7.7	0.00009	0.00180	1.98
Taf4a	-2.37	24.9	-5.0	0.00143	0.00858	-0.83

Sec24b	-2.36	26.1	-5.3	0.00098	0.00678	-0.44
Zzef1	-2.36	27.6	-4.8	0.00185	0.01019	-1.09
Rps15a	-2.36	29.2	-6.1	0.00045	0.00422	0.39
Dhrs9	-2.36	24.3	-4.1	0.00393	0.01695	-1.89
Cstf2	-2.36	26.1	-3.3	0.01283	0.03839	-3.11
Timm13	-2.36	25.5	-3.1	0.01762	0.04831	-3.44
Erc1	-2.36	24.8	-3.9	0.00528	0.02070	-2.19
Son	-2.36	26.9	-4.1	0.00407	0.01733	-1.91
Tnfaip8l2	-2.35	26.8	-3.2	0.01457	0.04201	-3.24
Myo1d	-2.35	24.2	-4.5	0.00254	0.01256	-1.43
Dip2b	-2.35	25.7	-3.6	0.00872	0.02924	-2.71
Tcerg1	-2.34	26.8	-6.6	0.00026	0.00308	0.96
Lsm2	-2.34	26.7	-4.1	0.00416	0.01759	-1.94
Nt5dc1	-2.34	25.3	-4.3	0.00311	0.01464	-1.64
Sec23ip	-2.34	25.9	-3.4	0.01061	0.03349	-2.91
Traf7	-2.33	25.1	-4.0	0.00453	0.01846	-2.04
Smg9	-2.33	25.0	-4.0	0.00459	0.01866	-2.05
Phpt1	-2.32	25.0	-3.8	0.00621	0.02310	-2.36
Acap2	-2.32	26.6	-4.3	0.00328	0.01501	-1.69
Psmc5	-2.32	28.6	-6.7	0.00023	0.00282	1.08
Arid1a	-2.31	29.9	-5.7	0.00065	0.00539	0.01
Ube2i	-2.31	26.9	-6.0	0.00046	0.00430	0.35
Gga1	-2.30	26.7	-4.5	0.00267	0.01291	-1.48
Cmip	-2.30	24.3	-4.9	0.00151	0.00892	-0.89
Kif2a	-2.30	25.3	-4.0	0.00445	0.01839	-2.02
Il1f9	-2.30	28.7	-5.1	0.00125	0.00791	-0.68
Nt5c2	-2.29	26.9	-5.0	0.00130	0.00808	-0.73
Prkab1	-2.29	24.7	-5.4	0.00082	0.00611	-0.25
Qdpr	-2.29	26.7	-6.2	0.00038	0.00378	0.54
Csnk2a1	-2.29	28.5	-7.3	0.00013	0.00215	1.63
Ppp1r12c	-2.29	25.0	-3.4	0.01053	0.03342	-2.91
Smarce1	-2.27	26.1	-4.3	0.00328	0.01501	-1.70
neo	-2.27	24.7	-3.4	0.01088	0.03408	-2.94
Psat1	-2.27	27.0	-4.5	0.00262	0.01278	-1.46
Ndufa8	-2.26	25.2	-3.8	0.00576	0.02199	-2.29
Uhrf1bp1l	-2.26	25.1	-3.0	0.01838	0.04992	-3.48
Gpcpd1	-2.26	24.2	-6.2	0.00038	0.00378	0.54
Caprin1	-2.26	25.6	-3.5	0.00935	0.03069	-2.78
Ptbp1	-2.26	30.7	-11.2	0.00001	0.00057	4.49
Ctps1	-2.26	24.5	-4.1	0.00439	0.01832	-2.00
Ubr2	-2.26	25.3	-5.2	0.00106	0.00706	-0.52
Arpc5	-2.25	31.0	-8.8	0.00004	0.00115	2.80
Aprt	-2.25	30.2	-11.6	0.00001	0.00048	4.71
Fkbp15	-2.24	28.5	-7.8	0.00008	0.00166	2.14
Nudcd2	-2.23	24.9	-3.7	0.00737	0.02591	-2.54
Ube2n	-2.23	28.0	-3.7	0.00741	0.02596	-2.54
Igbp1	-2.23	24.7	-4.3	0.00310	0.01463	-1.64
Rbck1	-2.22	24.0	-5.4	0.00083	0.00613	-0.26
Zc3h11a	-2.21	24.7	-3.0	0.01850	0.05000	-3.49
Smarcc1	-2.20	26.4	-3.8	0.00602	0.02261	-2.32
PTEN	-2.20	24.2	-5.1	0.00117	0.00758	-0.62
Psmc1	-2.19	28.0	-9.1	0.00003	0.00101	3.15
Ncor1	-2.19	28.7	-7.1	0.00016	0.00241	1.45
Cog1	-2.18	23.2	-3.4	0.01061	0.03349	-2.91
Ube2c	-2.18	24.0	-3.8	0.00629	0.02340	-2.37
Fgfr1op	-2.17	24.7	-3.7	0.00740	0.02596	-2.55
Sh2d3c	-2.17	24.4	-5.0	0.00135	0.00829	-0.77
Prmt5	-2.17	24.6	-5.4	0.00087	0.00627	-0.31
Ugdh	-2.17	27.9	-4.8	0.00184	0.01019	-1.08
Ywhah	-2.16	30.9	-8.4	0.00005	0.00130	2.55
Anks1	-2.16	25.1	-3.2	0.01341	0.03960	-3.16
Znf207	-2.16	27.0	-5.2	0.00105	0.00706	-0.51
Rpl7a	-2.16	28.6	-5.4	0.00090	0.00640	-0.33
Tuba8	-2.15	24.9	-3.7	0.00742	0.02597	-2.55

Trrap	-2.15	26.9	-4.2	0.00395	0.01699	-1.88
Guk1	-2.15	24.3	-4.5	0.00244	0.01221	-1.39
Myh10	-2.14	25.9	-3.8	0.00597	0.02248	-2.32
Mark2	-2.14	24.6	-4.5	0.00254	0.01256	-1.43
Gmfb	-2.14	26.3	-3.4	0.01131	0.03505	-2.98
Vps13b	-2.13	25.5	-4.3	0.00326	0.01501	-1.69
Larp4b	-2.13	25.1	-3.4	0.01138	0.03515	-2.99
Crocc	-2.12	27.5	-3.3	0.01292	0.03853	-3.12
Capg	-2.12	31.0	-12.5	0.00000	0.00045	5.16
Lrba	-2.12	25.5	-4.3	0.00299	0.01423	-1.60
Gyg	-2.11	29.4	-8.8	0.00004	0.00112	2.88
Smarcb1	-2.09	26.6	-3.4	0.01055	0.03342	-2.91
Psme3	-2.08	27.3	-6.3	0.00032	0.00350	0.72
Stam2	-2.08	24.9	-3.4	0.01106	0.03451	-2.96
Suds3	-2.08	23.9	-5.5	0.00075	0.00581	-0.16
Snx1	-2.08	27.7	-4.2	0.00375	0.01641	-1.83
Sbno1	-2.07	24.2	-3.1	0.01571	0.04431	-3.32
Eif5	-2.07	26.9	-4.7	0.00204	0.01096	-1.20
Dcp1a	-2.06	24.9	-3.7	0.00722	0.02556	-2.52
Flna	-2.06	37.2	-12.0	0.00000	0.00045	4.95
Uqcr10	-2.05	25.3	-3.2	0.01376	0.04048	-3.19
Ap1s2	-2.05	25.5	-5.0	0.00143	0.00861	-0.83
Eml3	-2.05	24.2	-4.6	0.00231	0.01189	-1.33
Pum2	-2.05	25.3	-4.0	0.00470	0.01892	-2.08
Cux1	-2.05	24.6	-3.8	0.00647	0.02371	-2.41
Ap3m1	-2.05	26.1	-5.0	0.00131	0.00810	-0.74
Ldha	-2.05	33.1	-8.6	0.00005	0.00120	2.69
Hdlbp	-2.04	27.3	-5.8	0.00057	0.00502	0.14
Rad23b	-2.04	27.0	-5.1	0.00120	0.00772	-0.64
Gmip	-2.04	28.6	-3.9	0.00567	0.02170	-2.26
Ppat	-2.04	24.5	-4.2	0.00384	0.01665	-1.86
Eif4h	-2.04	28.7	-7.4	0.00012	0.00200	1.74
Atn1	-2.03	24.2	-4.3	0.00324	0.01496	-1.69
Setd3	-2.03	25.2	-4.6	0.00232	0.01190	-1.34
Wnk1	-2.03	27.8	-5.3	0.00096	0.00668	-0.40
Bub3	-2.03	27.3	-3.4	0.01099	0.03439	-2.95
Rnaseh2a	-2.02	25.0	-4.4	0.00280	0.01347	-1.53
Stap1	-2.02	25.2	-4.7	0.00191	0.01048	-1.13
Pla2g4a	-2.02	26.8	-5.4	0.00087	0.00627	-0.31
Vrk1	-2.02	26.8	-3.9	0.00549	0.02131	-2.23
Npm1	-2.02	29.2	-4.3	0.00323	0.01494	-1.67
Ripk1	-2.02	26.4	-3.3	0.01320	0.03926	-3.14
Stfa2l1	-2.02	29.7	-8.8	0.00004	0.00112	2.90
Nup214	-2.02	27.9	-6.5	0.00026	0.00310	0.94
Akap13	-2.01	28.5	-3.9	0.00543	0.02120	-2.22
Map2k3	-2.01	26.8	-3.5	0.00924	0.03049	-2.77
Rbm3	-2.00	28.3	-5.3	0.00105	0.00706	-0.50
Eif4e2	-2.00	24.7	-3.8	0.00612	0.02292	-2.35
Cggbp1	-1.99	24.8	-3.9	0.00545	0.02124	-2.23
EG433182	-1.99	34.5	-9.0	0.00003	0.00106	3.03
Tpr	-1.98	32.4	-10.6	0.00001	0.00061	4.11
Gsdmdc1	-1.98	28.2	-4.1	0.00406	0.01733	-1.91
Usp34	-1.98	24.9	-3.5	0.00895	0.02975	-2.75
Exosc9	-1.98	24.8	-4.8	0.00167	0.00955	-0.99
Eps15l1	-1.97	27.5	-5.5	0.00075	0.00581	-0.15
Dpm1	-1.97	26.4	-5.7	0.00065	0.00539	0.00
Ktn1	-1.97	24.3	-4.1	0.00418	0.01763	-1.95
Psme4	-1.97	23.7	-3.9	0.00519	0.02043	-2.18
Spag9	-1.97	24.6	-3.2	0.01433	0.04162	-3.23
Rheb	-1.96	25.6	-3.4	0.00993	0.03216	-2.85
Prdx5	-1.96	33.6	-11.8	0.00000	0.00047	4.80
Gcc1	-1.96	25.1	-3.8	0.00620	0.02310	-2.36
Nit2	-1.96	24.4	-4.8	0.00175	0.00986	-1.04
Cic	-1.95	24.0	-3.2	0.01516	0.04317	-3.28

Ints2	-1.95	25.4	-4.0	0.00450	0.01845	-2.03
Rpsa	-1.95	30.4	-8.6	0.00004	0.00115	2.77
Snw1	-1.95	24.9	-3.8	0.00620	0.02310	-2.36
Galm	-1.95	25.6	-4.8	0.00170	0.00972	-1.01
Man2c1	-1.94	26.4	-4.5	0.00243	0.01220	-1.39
Pik3cg	-1.93	26.3	-3.5	0.00976	0.03178	-2.83
Dek	-1.93	27.5	-4.3	0.00335	0.01528	-1.71
Arhgdib	-1.93	34.6	-10.1	0.00001	0.00072	3.83
mKIAA0617	-1.93	28.1	-6.3	0.00032	0.00349	0.72
Ppp2r4	-1.92	28.4	-6.2	0.00036	0.00370	0.60
Dhx38	-1.92	25.5	-3.5	0.00907	0.03005	-2.76
Tceb1	-1.91	27.5	-5.7	0.00059	0.00505	0.09
Exosc6	-1.91	25.0	-4.6	0.00206	0.01104	-1.21
Dmxl2	-1.91	24.4	-3.3	0.01171	0.03601	-3.02
Dnajc8	-1.91	26.0	-3.6	0.00853	0.02888	-2.70
Coro1b	-1.91	28.6	-3.4	0.01109	0.03455	-2.96
Arhgap15	-1.90	24.5	-3.5	0.00984	0.03194	-2.84
Edc4	-1.90	28.2	-6.6	0.00024	0.00291	1.02
Hist1h2ah	-1.90	31.7	-3.0	0.01844	0.04997	-3.49
Zyx	-1.90	31.7	-9.0	0.00003	0.00106	3.05
GAPDH	-1.90	34.8	-11.2	0.00001	0.00057	4.47
Nup54	-1.89	27.7	-4.2	0.00374	0.01638	-1.83
Rint1	-1.89	23.5	-4.7	0.00185	0.01019	-1.10
Mtmr6	-1.89	24.5	-3.6	0.00851	0.02887	-2.69
Prkar2b	-1.88	25.6	-3.7	0.00662	0.02406	-2.43
Ehd1	-1.88	29.9	-6.9	0.00020	0.00268	1.21
Sec31a	-1.88	28.7	-7.0	0.00017	0.00247	1.36
Tubgcp2	-1.88	25.4	-3.1	0.01575	0.04431	-3.33
Mafg	-1.88	25.8	-3.8	0.00597	0.02248	-2.33
Naip2	-1.87	25.4	-3.0	0.01756	0.04818	-3.44
Tollip	-1.87	28.2	-7.0	0.00017	0.00246	1.36
Pnp	-1.87	32.5	-7.0	0.00018	0.00256	1.30
Gripap1	-1.86	28.6	-8.6	0.00004	0.00115	2.78
Tbcel	-1.86	24.0	-3.5	0.00866	0.02918	-2.71
Chd4	-1.86	29.9	-4.3	0.00322	0.01494	-1.67
Tpm4	-1.86	28.3	-6.4	0.00030	0.00334	0.80
Anxa1	-1.85	36.1	-9.9	0.00002	0.00076	3.71
Tmf1	-1.85	24.6	-3.2	0.01434	0.04163	-3.23
Myl6	-1.84	32.7	-9.9	0.00002	0.00076	3.68
Supt5h	-1.84	27.7	-7.0	0.00017	0.00247	1.35
Arpc1a	-1.84	24.9	-3.3	0.01276	0.03820	-3.11
Akr1a1	-1.84	27.9	-7.0	0.00017	0.00246	1.39
DXS254E	-1.84	24.8	-4.1	0.00436	0.01825	-2.00
Pebp1	-1.83	29.0	-8.4	0.00005	0.00128	2.59
Mapre1	-1.83	30.7	-5.4	0.00087	0.00627	-0.30
Hp1bp3	-1.83	28.4	-4.6	0.00230	0.01187	-1.32
Mdh1	-1.82	29.7	-3.8	0.00656	0.02395	-2.41
Akr1b3	-1.81	29.6	-9.4	0.00002	0.00087	3.36
Ccdc88b	-1.81	31.0	-9.1	0.00003	0.00102	3.12
Vasp	-1.81	31.0	-7.2	0.00015	0.00234	1.50
Tmod3	-1.80	29.2	-5.3	0.00100	0.00685	-0.44
Golgb1	-1.80	29.0	-5.2	0.00115	0.00754	-0.59
Rcsd1	-1.80	29.1	-5.9	0.00054	0.00488	0.20
Thop1	-1.80	26.8	-3.1	0.01595	0.04471	-3.34
Ncoa2	-1.79	24.3	-3.1	0.01660	0.04607	-3.38
Numb	-1.79	26.8	-3.8	0.00599	0.02255	-2.33
Acin1	-1.78	29.5	-3.3	0.01332	0.03952	-3.15
Macf1	-1.78	29.7	-5.2	0.00118	0.00766	-0.62
Cndp2	-1.78	28.5	-5.7	0.00065	0.00540	-0.01
Pfdn4	-1.77	24.5	-4.0	0.00472	0.01894	-2.08
Rfc4	-1.76	26.6	-4.8	0.00174	0.00983	-1.04
Rpl10a	-1.76	28.5	-7.2	0.00014	0.00218	1.59
Ssb	-1.76	27.5	-4.2	0.00372	0.01635	-1.82
Agfg1	-1.75	27.6	-6.7	0.00022	0.00277	1.11

Ndufa4	-1.75	26.1	-3.6	0.00796	0.02736	-2.62
Pfn1	-1.75	34.5	-9.1	0.00003	0.00102	3.13
Eps15	-1.75	27.8	-4.3	0.00339	0.01537	-1.72
Hmgb2	-1.75	30.6	-3.2	0.01475	0.04231	-3.25
Pkm	-1.75	34.1	-8.4	0.00005	0.00126	2.60
Pdcd10	-1.74	28.8	-7.5	0.00011	0.00193	1.83
Card9	-1.74	27.5	-4.1	0.00441	0.01832	-2.00
Urod	-1.73	24.2	-3.5	0.00892	0.02967	-2.74
Cops2	-1.73	26.0	-3.3	0.01271	0.03815	-3.11
Kmt2d	-1.73	25.9	-3.5	0.00952	0.03116	-2.81
Pcmt1	-1.73	27.5	-6.2	0.00038	0.00378	0.55
Map7d1	-1.73	24.3	-4.0	0.00442	0.01832	-2.01
Scfd2	-1.73	24.2	-3.8	0.00635	0.02348	-2.39
Apex1	-1.72	27.1	-3.2	0.01424	0.04147	-3.22
Psmb10	-1.71	27.9	-5.4	0.00086	0.00624	-0.30
Numa1	-1.71	27.2	-5.5	0.00074	0.00578	-0.14
Mpp1	-1.71	26.5	-3.9	0.00547	0.02126	-2.23
Pabpc4	-1.71	24.5	-4.1	0.00412	0.01747	-1.94
Cmpk1	-1.71	29.5	-7.4	0.00012	0.00197	1.77
Ep300	-1.70	25.8	-4.0	0.00451	0.01845	-2.03
Gdi2	-1.70	32.1	-8.7	0.00004	0.00113	2.86
Taf9	-1.69	24.2	-3.9	0.00566	0.02169	-2.27
Stxbp2	-1.69	29.4	-6.9	0.00018	0.00252	1.32
Psma2	-1.69	29.0	-6.8	0.00021	0.00269	1.16
Sec24d	-1.69	24.7	-3.2	0.01485	0.04254	-3.27
Znf830	-1.68	24.5	-3.7	0.00717	0.02546	-2.52
Add3	-1.68	28.8	-5.0	0.00139	0.00840	-0.79
Dusp6	-1.68	24.0	-4.6	0.00217	0.01144	-1.27
Plec	-1.67	34.4	-9.1	0.00003	0.00101	3.16
Necap2	-1.67	26.7	-4.9	0.00149	0.00883	-0.88
Mcm2	-1.66	28.5	-7.3	0.00012	0.00207	1.70
Hook3	-1.66	28.7	-6.8	0.00021	0.00269	1.16
Sae1	-1.65	27.7	-4.6	0.00210	0.01119	-1.23
Nrd1	-1.64	29.1	-3.7	0.00735	0.02591	-2.53
Lsp1	-1.64	32.2	-5.2	0.00114	0.00751	-0.59
C330027C09Rik	-1.64	25.5	-3.3	0.01157	0.03568	-3.01
Vbp1	-1.64	25.0	-3.4	0.01014	0.03268	-2.88
Ppp6r3	-1.64	27.7	-5.3	0.00091	0.00647	-0.36
Sf3a3	-1.64	29.2	-6.5	0.00027	0.00310	0.91
Eif4e	-1.64	27.3	-4.5	0.00235	0.01195	-1.35
Trip13	-1.63	26.1	-3.1	0.01617	0.04506	-3.36
Usp9x	-1.63	29.9	-7.3	0.00013	0.00210	1.66
Numa1	-1.63	33.2	-7.9	0.00008	0.00162	2.17
Copz1	-1.62	28.2	-6.3	0.00033	0.00355	0.69
Ap3b1	-1.62	28.1	-3.7	0.00695	0.02492	-2.47
Zbtb8os	-1.61	23.9	-3.1	0.01605	0.04496	-3.35
Phb2	-1.61	29.5	-6.1	0.00040	0.00388	0.50
Mtx2	-1.61	24.9	-3.1	0.01574	0.04431	-3.33
Unc119	-1.61	25.3	-3.2	0.01453	0.04193	-3.25
Mvp	-1.60	27.9	-4.8	0.00177	0.00992	-1.05
Acin1	-1.60	24.1	-3.1	0.01706	0.04713	-3.41
Lrrc59	-1.60	28.1	-3.9	0.00584	0.02227	-2.29
mCG_6739	-1.59	29.2	-6.2	0.00037	0.00372	0.59
Lrch4	-1.59	27.9	-3.8	0.00618	0.02310	-2.35
Vps8	-1.59	24.0	-4.5	0.00261	0.01274	-1.46
Nt5c	-1.59	27.4	-4.7	0.00185	0.01019	-1.10
Ptpn12	-1.59	28.2	-5.2	0.00110	0.00730	-0.55
S100a9	-1.59	37.5	-7.9	0.00008	0.00160	2.19
Flii	-1.58	28.8	-3.7	0.00745	0.02603	-2.55
Srsf2	-1.57	30.0	-4.5	0.00255	0.01258	-1.43
Snrnp40	-1.57	27.1	-3.7	0.00667	0.02420	-2.44
Scp2	-1.57	30.6	-5.1	0.00122	0.00782	-0.66
Ywhaz	-1.57	34.5	-9.8	0.00002	0.00076	3.64
Sf3a1	-1.57	29.9	-7.3	0.00013	0.00213	1.63

Eif3k	-1.57	27.7	-5.8	0.00058	0.00505	0.11
Pacsin2	-1.56	28.2	-5.2	0.00106	0.00709	-0.52
Fadd	-1.55	24.2	-3.7	0.00730	0.02577	-2.54
Rock1	-1.55	30.2	-6.7	0.00022	0.00274	1.13
Pygl	-1.55	33.2	-7.0	0.00017	0.00248	1.35
Lxn	-1.55	26.2	-3.4	0.01080	0.03391	-2.94
Utrn	-1.54	28.2	-4.2	0.00373	0.01638	-1.82
Apobr	-1.54	29.7	-3.7	0.00752	0.02622	-2.56
Eea1	-1.54	30.4	-4.9	0.00158	0.00921	-0.92
Acly	-1.53	29.2	-5.5	0.00080	0.00602	-0.22
Hnrnpab	-1.53	29.5	-6.3	0.00035	0.00361	0.64
Gstm1	-1.53	33.2	-8.3	0.00005	0.00130	2.55
Nfyc	-1.53	24.6	-3.3	0.01179	0.03617	-3.03
Ube2v1	-1.52	29.4	-4.3	0.00327	0.01501	-1.68
Fes	-1.52	27.5	-3.8	0.00655	0.02395	-2.41
Cab39	-1.51	28.7	-6.3	0.00033	0.00355	0.69
Nudt21	-1.51	28.4	-5.1	0.00116	0.00755	-0.61
Prdx6	-1.51	30.9	-5.6	0.00072	0.00571	-0.11
Usp14	-1.51	28.8	-7.0	0.00018	0.00249	1.33
Chchd3	-1.50	27.5	-4.0	0.00472	0.01895	-2.08
Ufd1l	-1.50	26.6	-3.7	0.00679	0.02445	-2.46
Ypel5	-1.50	24.7	-3.3	0.01314	0.03911	-3.14
Snx17	-1.49	24.4	-3.7	0.00724	0.02560	-2.53
Pgk1	-1.49	33.1	-6.3	0.00033	0.00355	0.70
Ptbp3	-1.49	29.7	-6.9	0.00018	0.00256	1.29
Ate1	-1.49	24.9	-3.6	0.00755	0.02629	-2.57
Psmc3	-1.48	29.0	-7.0	0.00017	0.00246	1.37
Hnrnpa2b1	-1.48	33.1	-8.3	0.00006	0.00133	2.50
Pmm2	-1.48	27.5	-4.9	0.00156	0.00912	-0.92
Rab11fip1	-1.47	27.8	-4.4	0.00297	0.01414	-1.59
Pgm2	-1.47	28.7	-6.2	0.00037	0.00375	0.58
Lamtor2	-1.46	27.6	-5.6	0.00066	0.00547	-0.03
Rbm27	-1.46	23.7	-3.7	0.00697	0.02498	-2.49
Sptbn1	-1.46	34.0	-8.9	0.00003	0.00108	2.99
Srp14	-1.45	24.6	-3.1	0.01572	0.04431	-3.33
Uba2	-1.45	27.4	-4.8	0.00176	0.00989	-1.05
Cherp	-1.45	27.9	-3.3	0.01233	0.03725	-3.07
Daglb	-1.45	26.2	-3.6	0.00853	0.02888	-2.70
Cbfb	-1.45	28.1	-4.6	0.00227	0.01173	-1.32
Psma1	-1.44	30.2	-8.1	0.00007	0.00150	2.32
Cstb	-1.43	27.9	-4.4	0.00289	0.01388	-1.57
Dazap1	-1.43	27.2	-3.4	0.01143	0.03528	-3.00
Rps28	-1.43	29.6	-3.6	0.00870	0.02923	-2.71
Mon2	-1.43	28.2	-6.5	0.00026	0.00310	0.93
Tpm3	-1.43	33.5	-3.3	0.01329	0.03946	-3.15
Ywhaq	-1.42	30.4	-7.0	0.00017	0.00246	1.38
Xpot	-1.42	26.6	-4.3	0.00335	0.01528	-1.72
Mettl9	-1.42	28.5	-5.2	0.00111	0.00734	-0.56
Rplp1	-1.42	27.9	-3.2	0.01389	0.04072	-3.19
Ube2r2	-1.42	24.8	-3.6	0.00859	0.02904	-2.70
Cct3	-1.42	30.2	-4.2	0.00363	0.01609	-1.80
Rpl11	-1.42	29.3	-4.6	0.00222	0.01155	-1.28
Rps20	-1.41	28.4	-5.6	0.00067	0.00549	-0.04
Tln1	-1.41	35.4	-8.3	0.00005	0.00130	2.54
Cast	-1.40	29.4	-5.3	0.00093	0.00657	-0.39
Prpf19	-1.40	28.8	-5.4	0.00084	0.00620	-0.28
Zc3h4	-1.40	28.2	-5.5	0.00075	0.00581	-0.15
Smndc1	-1.40	23.9	-3.3	0.01210	0.03692	-3.06
Pfdn1	-1.39	27.2	-4.9	0.00148	0.00880	-0.87
Sf3b5	-1.39	27.0	-4.2	0.00370	0.01628	-1.82
Rprd1b	-1.39	27.2	-3.1	0.01564	0.04426	-3.32
Rplp0	-1.39	30.2	-5.3	0.00096	0.00668	-0.41
Lsm8	-1.38	27.9	-4.8	0.00181	0.01008	-1.08
Ppp1r12a	-1.38	30.3	-5.1	0.00130	0.00808	-0.72

Rbx1	-1.37	26.8	-3.5	0.00977	0.03178	-2.84
Necap1	-1.36	24.1	-3.1	0.01619	0.04510	-3.36
Eif3c	-1.36	29.9	-7.0	0.00016	0.00243	1.41
Psmb2	-1.36	29.9	-6.3	0.00032	0.00351	0.71
Srrm2	-1.35	31.7	-4.7	0.00193	0.01057	-1.13
Ifi47	-1.35	28.4	-6.1	0.00041	0.00392	0.47
Atp6v1a	-1.35	31.3	-6.2	0.00038	0.00378	0.54
Sptan1	-1.35	34.7	-8.0	0.00007	0.00152	2.28
Syne2	-1.34	28.9	-4.6	0.00224	0.01161	-1.30
Ddx39b	-1.34	30.6	-4.0	0.00471	0.01892	-2.07
Tkt	-1.34	36.0	-5.3	0.00103	0.00697	-0.47
Cct2	-1.33	30.7	-6.2	0.00035	0.00365	0.62
Ppp1r18	-1.33	28.2	-5.1	0.00123	0.00782	-0.67
Ywhag	-1.32	31.5	-8.3	0.00006	0.00133	2.50
Fnbp1	-1.32	28.0	-5.7	0.00059	0.00505	0.09
Set	-1.32	28.7	-4.9	0.00146	0.00870	-0.85
Rab44	-1.31	30.4	-5.5	0.00080	0.00602	-0.23
Gpi	-1.31	34.6	-4.6	0.00239	0.01211	-1.36
Eif3f	-1.31	28.4	-5.6	0.00071	0.00567	-0.10
Wasf2	-1.31	28.8	-4.6	0.00216	0.01144	-1.26
Mthfd1	-1.31	28.9	-4.9	0.00160	0.00926	-0.95
Tpp2	-1.31	28.9	-3.7	0.00730	0.02577	-2.53
Arpc2	-1.30	32.1	-7.3	0.00013	0.00210	1.66
Pcna	-1.30	29.8	-6.3	0.00033	0.00355	0.70
Clic1	-1.29	32.2	-5.1	0.00131	0.00810	-0.73
Pnn	-1.29	27.7	-4.2	0.00344	0.01549	-1.75
Lrrfip1	-1.29	30.9	-5.3	0.00093	0.00657	-0.38
Snx2	-1.29	29.0	-4.0	0.00478	0.01912	-2.08
Xab2	-1.29	24.5	-3.4	0.01055	0.03342	-2.92
Tnpo3	-1.29	28.2	-4.5	0.00258	0.01265	-1.45
Cpne3	-1.29	32.4	-7.6	0.00010	0.00187	1.89
Mapk1	-1.28	28.8	-4.5	0.00247	0.01227	-1.40
Lasp1	-1.27	31.0	-4.3	0.00321	0.01494	-1.66
Dctn2	-1.27	28.5	-5.1	0.00118	0.00763	-0.63
Mki67	-1.27	29.6	-5.4	0.00087	0.00627	-0.31
Prpf8	-1.27	30.9	-7.1	0.00015	0.00234	1.50
Hnrnpk	-1.26	32.0	-7.1	0.00015	0.00234	1.50
Eef1b	-1.26	28.3	-4.3	0.00321	0.01494	-1.68
Anxa2	-1.26	33.8	-8.0	0.00007	0.00154	2.26
Capza1	-1.25	32.0	-5.2	0.00105	0.00706	-0.50
Rassf2	-1.25	28.5	-3.1	0.01748	0.04802	-3.43
Smarcc2	-1.25	29.8	-5.7	0.00059	0.00505	0.09
Rps9	-1.24	29.0	-5.6	0.00065	0.00540	-0.01
Atxn2l	-1.24	27.8	-3.8	0.00633	0.02347	-2.39
Mocos	-1.24	27.8	-4.2	0.00355	0.01577	-1.78
Fam129a	-1.24	30.5	-7.1	0.00016	0.00238	1.46
Ubr4	-1.23	29.3	-6.2	0.00038	0.00378	0.56
Hist1h1d	-1.23	33.3	-3.2	0.01465	0.04214	-3.25
Atic	-1.23	29.4	-4.5	0.00240	0.01211	-1.37
Pik3r1	-1.23	27.4	-3.4	0.01070	0.03367	-2.93
Actr1a	-1.23	28.2	-5.1	0.00125	0.00792	-0.69
Rnf20	-1.22	26.9	-3.7	0.00704	0.02515	-2.50
Psmb3	-1.22	29.1	-3.9	0.00550	0.02131	-2.23
Eif5a	-1.22	31.4	-3.6	0.00869	0.02923	-2.71
Tom1	-1.22	28.6	-4.3	0.00300	0.01423	-1.60
Tbc1d15	-1.22	27.7	-4.6	0.00219	0.01148	-1.28
Hnrnpa1	-1.21	30.5	-4.3	0.00346	0.01550	-1.75
Psmb8	-1.21	29.4	-4.5	0.00246	0.01225	-1.39
Coro1a	-1.21	34.6	-6.2	0.00038	0.00378	0.55
Apbb1ip	-1.21	29.3	-4.1	0.00450	0.01845	-2.02
Ugp2	-1.20	29.9	-6.6	0.00024	0.00289	1.03
Ostf1	-1.20	31.9	-4.5	0.00267	0.01291	-1.47
Mcm4	-1.20	28.1	-3.5	0.00939	0.03080	-2.79
Syk	-1.20	29.9	-3.7	0.00759	0.02640	-2.57

Pnkp	-1.20	31.3	-5.5	0.00075	0.00581	-0.15
Gdi1	-1.20	29.7	-5.7	0.00065	0.00540	-0.01
Pspc1	-1.19	27.2	-3.7	0.00677	0.02444	-2.46
Gcn1l1	-1.19	27.5	-3.2	0.01394	0.04082	-3.20
Srsf1	-1.19	29.6	-3.8	0.00604	0.02265	-2.33
Rps6ka1	-1.19	30.5	-3.0	0.01848	0.04999	-3.49
Rqcd1	-1.19	26.5	-3.4	0.01095	0.03428	-2.96
Dctn1	-1.19	29.3	-4.9	0.00156	0.00912	-0.92
Rps3a1	-1.19	30.3	-3.6	0.00799	0.02742	-2.62
Nup62	-1.18	26.9	-3.1	0.01621	0.04510	-3.36
Stk4	-1.18	29.6	-3.7	0.00682	0.02449	-2.45
Pgls	-1.18	30.6	-5.8	0.00054	0.00488	0.18
Arpc4	-1.16	33.1	-6.2	0.00038	0.00378	0.55
Gsn	-1.16	33.4	-7.1	0.00015	0.00235	1.48
Mcm6	-1.15	29.3	-5.2	0.00103	0.00697	-0.48
Eif2s1	-1.13	28.9	-3.6	0.00820	0.02803	-2.65
Slc9a3r1	-1.13	30.2	-5.4	0.00085	0.00620	-0.28
Tcp1	-1.13	30.6	-6.7	0.00023	0.00282	1.07
Rbbp4	-1.13	29.7	-5.5	0.00075	0.00582	-0.16
Blmh	-1.13	28.7	-3.3	0.01233	0.03725	-3.07
Plin3	-1.13	30.5	-5.8	0.00056	0.00499	0.14
Stk10	-1.12	29.1	-4.3	0.00308	0.01455	-1.63
Prep	-1.12	29.7	-5.6	0.00068	0.00550	-0.05
Anxa3	-1.12	32.8	-5.1	0.00125	0.00791	-0.69
Vps37b	-1.12	23.6	-3.0	0.01844	0.04997	-3.49
Ncf1	-1.11	32.2	-5.4	0.00082	0.00611	-0.25
Impdh2	-1.11	29.3	-6.3	0.00034	0.00357	0.66
Pdia3	-1.11	33.0	-4.4	0.00295	0.01408	-1.58
Pdia6	-1.11	30.5	-5.5	0.00079	0.00600	-0.21
Hnrnpd	-1.11	29.8	-4.4	0.00265	0.01286	-1.48
Ldhb	-1.10	28.4	-3.1	0.01632	0.04539	-3.36
Arcn1	-1.10	29.3	-5.8	0.00058	0.00505	0.12
Nup153	-1.10	28.2	-3.2	0.01480	0.04244	-3.26
Ncl	-1.10	31.1	-5.6	0.00071	0.00567	-0.10
Syncrip	-1.09	28.9	-5.8	0.00056	0.00499	0.15
Phb	-1.09	30.6	-4.0	0.00467	0.01884	-2.06
Hist1h1b	-1.09	31.2	-3.2	0.01398	0.04087	-3.20
Nono	-1.09	29.6	-4.2	0.00379	0.01654	-1.85
Birc6	-1.08	26.8	-3.1	0.01732	0.04771	-3.43
Acbd3	-1.08	27.6	-3.9	0.00506	0.02003	-2.15
Psmd13	-1.08	29.6	-5.5	0.00074	0.00578	-0.14
Flnb	-1.08	31.0	-5.2	0.00105	0.00706	-0.51
Sf1	-1.07	28.8	-3.1	0.01707	0.04713	-3.41
Raly	-1.07	29.2	-3.7	0.00706	0.02520	-2.49
PSME2b	-1.07	30.1	-3.6	0.00870	0.02923	-2.71
Cltc	-1.07	34.4	-5.0	0.00135	0.00829	-0.77
Bag6	-1.07	28.8	-4.3	0.00317	0.01481	-1.66
Gstp1	-1.07	30.0	-4.7	0.00195	0.01063	-1.15
Mcm5	-1.07	28.8	-4.2	0.00342	0.01540	-1.74
Fen1	-1.06	28.2	-3.8	0.00593	0.02242	-2.32
Elavl1	-1.06	30.5	-3.8	0.00662	0.02406	-2.42
Pcbp2	-1.06	29.7	-5.0	0.00138	0.00840	-0.80
Vat1	-1.06	30.2	-4.5	0.00234	0.01195	-1.35
Api5	-1.06	28.6	-3.1	0.01546	0.04381	-3.30
Ap3s1	-1.05	27.7	-3.3	0.01217	0.03711	-3.06
Npepps	-1.05	30.7	-4.5	0.00254	0.01255	-1.43
Psma3	-1.05	30.9	-5.0	0.00134	0.00826	-0.76
Cfl1	-1.05	34.4	-3.1	0.01608	0.04499	-3.34
Ncapd2	-1.05	28.8	-4.2	0.00365	0.01615	-1.81
Mapre2	-1.04	28.2	-3.4	0.01049	0.03342	-2.91
Fasn	-1.04	27.8	-3.5	0.00989	0.03206	-2.85
Cbl	-1.04	30.8	-5.7	0.00060	0.00507	0.08
Appl1	-1.04	27.5	-4.1	0.00395	0.01699	-1.89
Padi4	-1.04	30.9	-4.9	0.00147	0.00874	-0.86

Plcg2	-1.03	31.1	-4.1	0.00440	0.01832	-2.00
Cct6a	-1.03	30.4	-5.0	0.00131	0.00810	-0.74
Nup188	-1.03	27.9	-4.3	0.00320	0.01494	-1.67
Ywhae	-1.03	32.4	-5.8	0.00054	0.00488	0.18
Ankrd13a	-1.03	27.9	-3.9	0.00532	0.02082	-2.20
Snrnp70	-1.02	27.7	-3.3	0.01247	0.03756	-3.09
Tsg101	-1.02	27.1	-3.2	0.01407	0.04108	-3.21
Dock8	-1.02	31.1	-5.4	0.00083	0.00613	-0.27
Hnrnpu	-1.01	31.8	-3.7	0.00681	0.02449	-2.45
Lamtor1	-1.01	27.1	-3.1	0.01587	0.04455	-3.34
Dock11	-1.00	29.3	-3.5	0.00953	0.03117	-2.80
Fmnl1	-1.00	31.0	-4.3	0.00325	0.01497	-1.69
Msn	-1.00	33.8	-5.6	0.00067	0.00549	-0.04
Psmb1	-1.00	30.6	-5.5	0.00080	0.00602	-0.22
Nup205	-1.00	30.2	-5.3	0.00100	0.00684	-0.45
Add1	-0.99	30.1	-4.6	0.00206	0.01104	-1.21
Casp8	-0.99	28.0	-3.6	0.00838	0.02854	-2.68
Hdac1	-0.99	28.8	-4.6	0.00220	0.01149	-1.28
Rock2	-0.98	28.9	-5.2	0.00113	0.00743	-0.58
Vim	-0.98	35.6	-3.8	0.00644	0.02366	-2.40
Pxn	-0.98	29.4	-3.6	0.00763	0.02649	-2.58
Rbm12	-0.97	27.8	-3.7	0.00713	0.02534	-2.51
Myh9	-0.97	37.4	-5.4	0.00085	0.00620	-0.29
Vps35	-0.97	31.0	-4.2	0.00352	0.01569	-1.77
Elmo1	-0.97	30.7	-3.5	0.00976	0.03178	-2.83
Psma5	-0.95	30.4	-3.4	0.01032	0.03308	-2.89
Itgal	-0.95	30.2	-4.4	0.00264	0.01283	-1.47
Ppp6r1	-0.95	28.7	-4.0	0.00469	0.01888	-2.07
Cotl1	-0.94	33.3	-4.1	0.00388	0.01673	-1.87
Pcyt1a	-0.94	28.2	-4.2	0.00352	0.01569	-1.77
Atrx	-0.94	27.4	-3.2	0.01493	0.04264	-3.28
Fbl	-0.94	27.8	-3.4	0.01075	0.03377	-2.94
Cpne1	-0.93	30.0	-4.4	0.00270	0.01301	-1.50
Tardbp	-0.92	30.6	-4.2	0.00347	0.01551	-1.76
Uso1	-0.92	29.3	-4.0	0.00486	0.01941	-2.11
Hnrnpm	-0.91	32.8	-3.8	0.00640	0.02353	-2.39
Anxa7	-0.91	30.3	-4.5	0.00260	0.01271	-1.46
Xpnpep1	-0.91	28.6	-4.3	0.00300	0.01423	-1.61
Kars	-0.91	28.6	-4.4	0.00285	0.01367	-1.55
Ahsa1	-0.91	28.8	-4.5	0.00257	0.01263	-1.44
Vps13c	-0.91	29.4	-4.0	0.00460	0.01868	-2.05
Tubb5	-0.91	32.4	-3.5	0.00954	0.03118	-2.80
Picalm	-0.90	29.7	-5.0	0.00139	0.00840	-0.80
Hspa8	-0.90	34.9	-5.6	0.00069	0.00560	-0.07
Myo18a	-0.89	29.2	-4.3	0.00328	0.01501	-1.70
Mapk14	-0.89	29.6	-3.4	0.01052	0.03342	-2.91
Wdfy4	-0.88	29.3	-3.7	0.00672	0.02430	-2.45
Rdx	-0.88	29.8	-3.7	0.00680	0.02446	-2.46
Tubb4b	-0.88	33.8	-4.6	0.00217	0.01144	-1.27
Eprs	-0.88	29.6	-4.0	0.00448	0.01843	-2.02
Lta4h	-0.88	35.2	-4.8	0.00181	0.01008	-1.08
Cct4	-0.87	30.6	-3.6	0.00773	0.02677	-2.59
Psmd1	-0.87	30.5	-3.9	0.00547	0.02126	-2.23
Gak	-0.87	27.9	-3.7	0.00710	0.02526	-2.50
Fubp1	-0.87	28.9	-3.3	0.01209	0.03691	-3.06
Golga2	-0.87	28.4	-3.1	0.01536	0.04358	-3.31
Idh1	-0.87	30.6	-5.4	0.00081	0.00610	-0.24
Rgs14	-0.87	28.2	-3.6	0.00811	0.02778	-2.64
Taldo1	-0.87	33.7	-3.9	0.00551	0.02132	-2.24
Cnot1	-0.87	30.1	-4.6	0.00212	0.01129	-1.24
Pgd	-0.87	34.1	-4.3	0.00312	0.01464	-1.65
Cap1	-0.86	34.2	-3.5	0.00884	0.02952	-2.73
Gca	-0.86	29.8	-4.5	0.00241	0.01213	-1.38
Snd1	-0.86	30.5	-3.9	0.00556	0.02147	-2.25

Hsp90aa1	-0.85	32.8	-5.4	0.00082	0.00611	-0.25
Msra	-0.85	31.5	-4.8	0.00179	0.01003	-1.07
Lmnb2	-0.85	31.0	-3.2	0.01526	0.04338	-3.29
Psmd2	-0.85	30.1	-4.0	0.00440	0.01832	-2.01
Casp3	-0.85	28.0	-3.1	0.01552	0.04395	-3.32
Mtpn	-0.84	29.6	-4.6	0.00207	0.01105	-1.22
Park7	-0.84	30.4	-3.9	0.00515	0.02030	-2.17
Anxa5	-0.84	31.2	-5.1	0.00122	0.00781	-0.66
Ranbp3	-0.82	28.2	-3.8	0.00600	0.02256	-2.33
Eef1d	-0.82	30.9	-4.3	0.00336	0.01531	-1.73
Tpi1	-0.82	32.6	-3.6	0.00840	0.02858	-2.68
Huwe1	-0.82	28.7	-3.2	0.01389	0.04072	-3.20
Hspa4	-0.81	30.8	-4.6	0.00215	0.01141	-1.26
Ddb1	-0.81	31.0	-3.9	0.00568	0.02172	-2.27
Anxa11	-0.80	32.5	-5.0	0.00131	0.00810	-0.74
Dbnl	-0.80	31.0	-4.5	0.00242	0.01218	-1.38
Psmd12	-0.80	29.2	-4.0	0.00446	0.01840	-2.02
Vta1	-0.79	30.0	-3.1	0.01735	0.04773	-3.43
Tsta3	-0.79	28.5	-3.2	0.01371	0.04039	-3.19
Myo1f	-0.79	31.0	-3.4	0.01018	0.03275	-2.88
Adprh	-0.79	29.0	-3.4	0.01102	0.03442	-2.96
Il16	-0.79	31.8	-4.6	0.00221	0.01155	-1.29
Psmd5	-0.79	29.1	-3.4	0.01001	0.03235	-2.86
Apaf1	-0.78	29.2	-3.2	0.01442	0.04174	-3.24
Clip1	-0.78	30.3	-4.1	0.00403	0.01723	-1.91
Eif3m	-0.78	28.0	-3.0	0.01837	0.04992	-3.49
Psmb4	-0.77	29.5	-3.8	0.00608	0.02278	-2.34
Smc3	-0.76	30.5	-4.4	0.00265	0.01286	-1.48
Nup155	-0.76	29.9	-3.8	0.00586	0.02229	-2.30
Pdcd6ip	-0.73	31.4	-3.8	0.00593	0.02242	-2.32
Nhp2l1	-0.73	28.7	-3.4	0.01039	0.03321	-2.90
Psme1	-0.73	31.4	-3.7	0.00722	0.02556	-2.52
Syne1	-0.72	33.7	-3.4	0.01117	0.03475	-2.98
Pgm2	-0.72	30.4	-4.5	0.00235	0.01195	-1.35
Psmc4	-0.71	29.8	-3.4	0.01129	0.03505	-2.99
Mapk3	-0.71	30.5	-3.9	0.00531	0.02081	-2.20
Dock2	-0.71	31.7	-3.1	0.01610	0.04502	-3.35
Vcp	-0.71	32.4	-4.2	0.00355	0.01577	-1.78
Pkn1	-0.70	28.3	-3.0	0.01831	0.04989	-3.49
Ppp1r7	-0.70	28.8	-3.6	0.00817	0.02795	-2.65
Rac2	-0.69	34.0	-3.4	0.01061	0.03349	-2.92
Psma7	-0.68	30.9	-4.1	0.00420	0.01769	-1.96
Xpo7	-0.68	28.8	-3.5	0.00917	0.03026	-2.77
Xpo1	-0.68	30.7	-3.8	0.00590	0.02237	-2.31
Rrbp1	-0.67	31.2	-3.1	0.01530	0.04344	-3.30
Usp5	-0.66	30.6	-4.2	0.00378	0.01649	-1.85
Inpp5d	-0.66	29.6	-3.1	0.01580	0.04439	-3.33
Txn	-0.65	33.2	-4.0	0.00500	0.01984	-2.14
Eif3l	-0.65	30.7	-3.7	0.00712	0.02531	-2.51
Pa2g4	-0.64	30.4	-3.3	0.01247	0.03756	-3.09
Arhgap25	-0.64	30.1	-3.4	0.01082	0.03393	-2.94
Myo1g	-0.64	30.1	-3.3	0.01231	0.03725	-3.08
Smc2	-0.62	29.7	-3.7	0.00699	0.02499	-2.49
Eftud2	-0.62	30.3	-3.3	0.01287	0.03845	-3.12
Ncf2	-0.62	32.4	-3.3	0.01223	0.03718	-3.07
Arfgef1	-0.61	29.4	-3.3	0.01286	0.03844	-3.12
Ppp2r1a	-0.60	31.2	-3.2	0.01436	0.04164	-3.24
Coro7	-0.59	31.3	-3.1	0.01566	0.04427	-3.32
Sf3b3	-0.59	30.7	-3.7	0.00724	0.02560	-2.53
Copb2	-0.59	30.3	-3.2	0.01469	0.04221	-3.26
G6pdx	-0.59	34.5	-3.1	0.01578	0.04437	-3.33
Erh	-0.59	30.5	-3.2	0.01410	0.04114	-3.22
Sh3bgrl	-0.58	29.2	-3.2	0.01387	0.04072	-3.20
Actn1	-0.58	35.4	-3.4	0.01013	0.03268	-2.87

Copb1	-0.55	30.7	-3.3	0.01163	0.03584	-3.02
Hnrnpl	-0.52	30.8	-3.3	0.01263	0.03796	-3.10
Eef2	-0.52	33.6	-3.3	0.01185	0.03627	-3.04
Actn4	-0.51	33.6	-3.3	0.01221	0.03717	-3.07
Arpc3	-0.49	31.9	-3.1	0.01614	0.04504	-3.36
Rab8a	0.62	29.5	3.3	0.01176	0.03611	-3.03
Dcps	0.67	29.4	3.7	0.00678	0.02445	-2.46
Slc27a4	0.67	28.1	3.0	0.01828	0.04983	-3.48
Ap2m1	0.68	28.4	3.4	0.01053	0.03342	-2.92
Ptpn11	0.69	28.8	3.6	0.00763	0.02649	-2.58
Ap2s1	0.70	29.0	3.2	0.01429	0.04153	-3.23
Fis1	0.70	30.7	4.2	0.00380	0.01655	-1.85
Gm5483	0.71	31.1	4.0	0.00494	0.01966	-2.13
Nckap1l	0.72	30.2	3.3	0.01165	0.03586	-3.02
Hpcal1	0.72	30.1	3.7	0.00671	0.02430	-2.45
Ada	0.72	32.1	3.7	0.00709	0.02526	-2.50
Sod1	0.72	30.1	3.4	0.01123	0.03491	-2.98
Txnrd1	0.74	31.1	4.0	0.00455	0.01853	-2.04
Gsr	0.78	34.1	4.1	0.00424	0.01781	-1.97
Twf2	0.80	32.9	3.4	0.01055	0.03342	-2.91
Pstpip1	0.80	28.9	3.4	0.01066	0.03360	-2.93
Ap1b1	0.82	30.4	3.7	0.00657	0.02395	-2.43
Naga	0.82	28.7	3.1	0.01706	0.04713	-3.41
Glo1	0.82	28.7	4.1	0.00438	0.01831	-2.00
Cbx1	0.84	28.2	3.2	0.01463	0.04211	-3.25
Selenbp1	0.84	30.8	4.8	0.00182	0.01010	-1.08
Nek9	0.84	27.4	3.0	0.01842	0.04997	-3.49
Supt16	0.87	28.5	3.3	0.01262	0.03793	-3.10
Bax	0.87	29.0	4.0	0.00452	0.01845	-2.03
Srsf3	0.88	32.1	3.8	0.00619	0.02310	-2.36
Fgr	0.88	31.0	4.5	0.00236	0.01196	-1.35
Plaur	0.88	27.6	3.3	0.01273	0.03818	-3.11
Mad2l1	0.89	29.2	4.0	0.00495	0.01966	-2.13
Hrsp12	0.89	27.9	3.1	0.01615	0.04504	-3.36
Actg1	0.90	35.4	5.0	0.00142	0.00854	-0.82
Slc35b2	0.90	28.2	3.2	0.01507	0.04297	-3.29
Esyt1	0.91	30.3	5.1	0.00124	0.00791	-0.69
Ppp5c	0.92	27.6	3.2	0.01508	0.04297	-3.29
Pwp1	0.92	27.7	3.3	0.01229	0.03725	-3.07
Tm9sf3	0.92	29.8	3.0	0.01833	0.04989	-3.48
Arid3a	0.93	28.4	4.1	0.00411	0.01745	-1.93
Nucb2	0.94	27.5	3.1	0.01613	0.04504	-3.36
Pitpna	0.95	30.6	3.6	0.00867	0.02922	-2.70
Rap1b	0.95	31.8	3.4	0.01113	0.03465	-2.96
Lyn	0.96	30.7	4.5	0.00261	0.01273	-1.46
Por	0.96	28.7	3.4	0.01105	0.03451	-2.97
Srpk2	0.97	27.9	3.4	0.01027	0.03295	-2.89
Rab10	0.98	29.8	4.9	0.00162	0.00934	-0.96
Tmem2	0.98	29.6	4.6	0.00222	0.01155	-1.29
Rab21	0.99	30.0	5.2	0.00113	0.00743	-0.58
Usp7	1.00	30.7	4.8	0.00166	0.00953	-0.99
Anxa4	1.00	28.3	3.6	0.00780	0.02693	-2.60
Ccar2	1.00	28.7	4.9	0.00158	0.00921	-0.93
Rpia	1.01	31.8	5.7	0.00059	0.00505	0.09
Plcl2	1.01	28.8	4.2	0.00367	0.01621	-1.82
Hnrnph2	1.01	27.9	4.3	0.00335	0.01528	-1.72
Ano6	1.02	28.4	3.5	0.00983	0.03194	-2.84
Pepd	1.02	28.7	3.4	0.01134	0.03509	-2.99
Sorl1	1.03	30.4	5.8	0.00058	0.00505	0.10
Man2b1	1.04	31.3	5.6	0.00072	0.00572	-0.12
Gm20425	1.04	30.5	4.2	0.00386	0.01671	-1.87
Wbp2	1.04	27.9	3.5	0.00872	0.02924	-2.72
Naaa	1.05	27.3	3.3	0.01188	0.03634	-3.04
Snrpd2	1.05	30.7	4.2	0.00387	0.01671	-1.87

Csrp1	1.05	29.5	3.2	0.01340	0.03960	-3.16
Trem3	1.06	27.7	3.9	0.00524	0.02059	-2.19
Ctsz	1.08	30.5	4.7	0.00195	0.01063	-1.16
Nras	1.08	27.8	4.6	0.00219	0.01149	-1.28
Cpd	1.09	26.9	3.4	0.01034	0.03311	-2.90
Man2b2	1.09	27.2	3.8	0.00656	0.02395	-2.42
Golt1b	1.09	27.3	3.9	0.00510	0.02013	-2.16
Mfsd1	1.11	27.1	3.4	0.01025	0.03290	-2.89
Syngr2	1.12	30.0	3.6	0.00793	0.02732	-2.61
Got1	1.12	31.2	6.5	0.00028	0.00322	0.85
Rab27a	1.13	30.1	4.3	0.00323	0.01494	-1.67
Ceacam1	1.13	29.3	4.1	0.00406	0.01733	-1.92
Slc15a4	1.13	26.0	3.0	0.01825	0.04979	-3.48
Ddost	1.14	29.1	5.3	0.00098	0.00678	-0.44
Pzp	1.15	28.2	3.5	0.00998	0.03229	-2.86
Lrp1	1.15	28.6	3.6	0.00773	0.02677	-2.59
Ssr4	1.15	29.0	4.0	0.00452	0.01846	-2.03
Prkcsh	1.15	29.4	3.3	0.01176	0.03611	-3.02
Rab4b	1.15	28.4	5.3	0.00092	0.00655	-0.38
Smpdl3a	1.16	28.8	5.8	0.00057	0.00505	0.13
Gss	1.16	26.6	3.4	0.01055	0.03342	-2.92
Itgb1	1.16	29.0	3.8	0.00636	0.02348	-2.38
Rab35	1.16	28.5	3.9	0.00557	0.02147	-2.25
Eif2b5	1.18	26.7	3.1	0.01749	0.04802	-3.44
Rap2c	1.18	27.9	4.7	0.00195	0.01063	-1.16
Gp1ba	1.20	26.5	3.1	0.01595	0.04471	-3.34
Atp6v0a2	1.20	26.5	3.5	0.00915	0.03024	-2.77
Pithd1	1.21	26.4	3.2	0.01395	0.04082	-3.21
Etfa	1.21	28.6	3.1	0.01680	0.04653	-3.39
Sf3b6	1.22	27.8	3.6	0.00860	0.02904	-2.70
U2surp	1.22	28.2	3.3	0.01233	0.03725	-3.07
Slc9a6	1.22	25.3	3.1	0.01736	0.04773	-3.43
Atp2a3	1.22	32.8	4.0	0.00467	0.01884	-2.06
Stx4	1.23	26.9	3.8	0.00632	0.02346	-2.38
Serpina1d	1.23	28.2	4.2	0.00345	0.01549	-1.75
Cd82	1.24	27.9	4.7	0.00197	0.01068	-1.17
Tmed9	1.25	29.3	6.8	0.00021	0.00269	1.16
Snrpd1	1.25	31.1	3.4	0.01017	0.03273	-2.87
Slc16a1	1.25	28.7	4.3	0.00341	0.01540	-1.74
Alb	1.26	33.2	4.6	0.00240	0.01213	-1.36
Atp1b3	1.27	28.9	6.0	0.00047	0.00431	0.33
Sypl	1.27	29.3	3.6	0.00811	0.02778	-2.63
Gosr1	1.28	26.7	3.7	0.00737	0.02591	-2.54
Tmed7	1.28	27.7	3.8	0.00586	0.02229	-2.31
Tbc1d17	1.28	26.0	3.1	0.01523	0.04334	-3.30
Cd97	1.28	30.7	7.5	0.00011	0.00194	1.80
Snrpn	1.29	30.2	6.6	0.00024	0.00288	1.04
Tmem63a	1.30	30.0	6.0	0.00046	0.00431	0.34
Prdx2	1.30	31.6	4.8	0.00174	0.00983	-1.03
Manf	1.30	29.4	6.2	0.00036	0.00370	0.59
Serpinb1a	1.31	35.4	4.1	0.00449	0.01845	-2.02
Entpd1	1.31	26.9	3.4	0.01012	0.03267	-2.87
Ttc38	1.31	26.8	3.8	0.00637	0.02348	-2.39
Rtn3	1.31	30.0	3.4	0.01074	0.03377	-2.93
Vkorc1l1	1.31	24.6	3.2	0.01489	0.04258	-3.27
Cycs	1.32	30.9	4.5	0.00258	0.01265	-1.44
Fcn2	1.33	27.9	5.5	0.00073	0.00576	-0.13
Esyt2	1.33	27.1	4.1	0.00415	0.01759	-1.95
Glg1	1.33	30.9	5.0	0.00135	0.00829	-0.76
Ralb	1.35	29.2	5.7	0.00062	0.00522	0.04
Alcam	1.35	23.5	3.4	0.01131	0.03505	-2.99
Adam10	1.36	28.7	5.7	0.00059	0.00505	0.10
Vcam1	1.36	27.6	4.5	0.00246	0.01225	-1.40
Lilrb3	1.37	28.7	6.6	0.00024	0.00288	1.04

Atp2a2	1.37	30.2	5.8	0.00056	0.00497	0.15
Serpina1c	1.38	30.3	4.8	0.00172	0.00980	-1.02
Emb	1.38	27.8	4.0	0.00476	0.01907	-2.09
Arsb	1.38	30.1	6.7	0.00021	0.00273	1.14
Mgam	1.40	30.4	8.0	0.00007	0.00155	2.24
env	1.40	27.6	3.8	0.00639	0.02350	-2.39
Rab5b	1.43	29.0	7.8	0.00009	0.00174	2.07
Rab7	1.43	32.8	7.6	0.00009	0.00180	1.97
Tra2b	1.44	30.9	6.0	0.00043	0.00412	0.41
Npl	1.45	27.5	5.3	0.00098	0.00678	-0.44
Emd	1.45	27.6	4.8	0.00164	0.00944	-0.97
Cd9	1.45	31.2	5.2	0.00117	0.00761	-0.61
Pld3	1.46	29.2	4.1	0.00424	0.01781	-1.96
Igkc	1.46	28.7	5.2	0.00113	0.00743	-0.58
Cd63	1.47	28.8	6.5	0.00028	0.00318	0.88
Foxk1	1.48	25.9	3.8	0.00591	0.02237	-2.31
Clcn3	1.48	23.8	3.0	0.01772	0.04851	-3.45
Gpx1	1.48	33.0	8.8	0.00004	0.00112	2.89
Lman1	1.48	28.8	3.3	0.01307	0.03893	-3.13
Tmed10	1.49	29.7	4.6	0.00223	0.01156	-1.28
Atp6ap1	1.49	26.9	4.2	0.00384	0.01666	-1.87
Plvap	1.49	24.8	3.5	0.00932	0.03063	-2.79
Ddx60	1.49	25.8	3.3	0.01195	0.03652	-3.05
H6pd	1.49	29.3	6.1	0.00039	0.00384	0.51
Tomm70a	1.50	27.3	5.4	0.00089	0.00634	-0.33
Acp2	1.50	26.3	3.6	0.00799	0.02742	-2.63
Acsl5	1.50	26.7	4.3	0.00324	0.01496	-1.69
Man1c1	1.50	24.8	3.5	0.00890	0.02962	-2.74
Mmrn1	1.51	27.8	3.4	0.01059	0.03349	-2.91
Itga4	1.51	25.6	3.2	0.01460	0.04207	-3.25
Itgb2	1.52	34.9	3.5	0.00888	0.02960	-2.73
Arl8a	1.53	28.4	6.6	0.00026	0.00309	0.95
Ppih	1.54	28.2	4.2	0.00384	0.01665	-1.85
Psd4	1.54	26.9	5.0	0.00140	0.00844	-0.81
Oas3	1.54	28.2	3.7	0.00673	0.02434	-2.44
Hmbs	1.55	28.3	5.5	0.00079	0.00600	-0.21
Dld	1.56	30.0	5.4	0.00088	0.00633	-0.32
Rab8b	1.57	29.1	4.1	0.00417	0.01760	-1.94
Myom1	1.58	27.8	3.2	0.01339	0.03960	-3.16
Slc1a5	1.58	25.7	3.7	0.00671	0.02430	-2.45
Ifitm2	1.58	28.1	3.9	0.00562	0.02164	-2.25
Plscr1	1.58	28.5	5.3	0.00093	0.00655	-0.38
Abhd12	1.59	24.0	3.5	0.00884	0.02952	-2.73
Apob	1.59	26.8	3.4	0.00997	0.03228	-2.86
Hpx	1.61	29.4	7.6	0.00010	0.00183	1.93
Clca1	1.61	24.1	3.1	0.01674	0.04639	-3.39
Man2a1	1.62	30.2	8.5	0.00005	0.00120	2.70
Brd3	1.62	26.6	4.3	0.00308	0.01455	-1.63
Ormdl2	1.62	25.4	3.9	0.00564	0.02165	-2.27
Rab32	1.62	30.1	7.6	0.00010	0.00181	1.95
C5ar1	1.63	30.8	4.3	0.00338	0.01534	-1.72
Tor1aip1	1.64	27.1	4.4	0.00293	0.01402	-1.58
Itga2b	1.64	30.6	6.4	0.00032	0.00349	0.74
Lman2	1.64	28.9	6.8	0.00021	0.00269	1.18
B2m	1.65	28.6	3.3	0.01226	0.03725	-3.06
Emr1	1.67	25.3	3.2	0.01493	0.04264	-3.28
Tmem30a	1.68	28.4	7.5	0.00011	0.00193	1.82
Golga7	1.69	26.2	3.2	0.01426	0.04148	-3.23
Cpox	1.69	27.7	6.3	0.00034	0.00357	0.67
Ifitm3	1.70	31.0	7.1	0.00016	0.00241	1.44
Slc40a1	1.70	27.4	4.7	0.00185	0.01019	-1.10
Tmem43	1.70	28.5	3.9	0.00553	0.02136	-2.23
Slc44a2	1.70	29.1	5.5	0.00083	0.00613	-0.25
Rab6a	1.70	31.9	8.8	0.00004	0.00112	2.93

Rab3d	1.72	30.4	7.0	0.00017	0.00246	1.37
Ndst1	1.73	26.1	3.5	0.00881	0.02946	-2.73
Nolc1	1.73	27.3	4.0	0.00510	0.02013	-2.15
Fpr2	1.74	30.2	5.0	0.00147	0.00874	-0.85
Ctse	1.74	29.4	5.4	0.00094	0.00658	-0.38
Rab14	1.75	31.8	9.6	0.00002	0.00084	3.48
M6pr	1.75	28.8	7.8	0.00008	0.00170	2.10
Rnaset2b	1.76	26.4	4.3	0.00315	0.01476	-1.66
Rab5a	1.77	28.1	7.3	0.00012	0.00208	1.69
Rab18	1.77	28.0	7.3	0.00013	0.00209	1.68
Ctnnb1	1.79	26.3	4.8	0.00166	0.00954	-0.99
Mgst1	1.80	29.3	3.4	0.01070	0.03367	-2.92
Gns	1.81	28.7	6.1	0.00040	0.00390	0.49
Atxn3	1.81	24.4	4.2	0.00345	0.01549	-1.75
Nhlrc3	1.81	26.8	6.0	0.00047	0.00431	0.33
Gusb	1.82	32.0	7.5	0.00011	0.00193	1.83
Prdx4	1.82	30.3	10.7	0.00001	0.00057	4.21
Glb1	1.82	30.6	6.0	0.00046	0.00429	0.37
Tgfbi	1.84	26.4	4.7	0.00195	0.01063	-1.16
Ctsa	1.84	28.7	5.6	0.00072	0.00571	-0.10
Rab1b	1.84	30.7	9.7	0.00002	0.00081	3.54
F10	1.85	24.9	4.4	0.00282	0.01357	-1.54
Serpinc1	1.86	27.0	5.6	0.00067	0.00549	-0.04
Rnf130	1.86	23.9	4.6	0.00232	0.01190	-1.34
Abcg2	1.87	26.0	3.2	0.01470	0.04221	-3.26
Dad1	1.87	28.4	3.9	0.00564	0.02165	-2.26
Ptgir	1.87	24.1	3.7	0.00661	0.02406	-2.43
Ctsd	1.88	31.5	6.9	0.00020	0.00268	1.21
Slc28a2	1.88	30.4	7.6	0.00010	0.00189	1.88
Reep5	1.88	28.9	3.7	0.00748	0.02610	-2.55
Snrpd3	1.88	31.3	9.8	0.00002	0.00076	3.62
Pgrmc2	1.89	25.5	3.7	0.00657	0.02395	-2.42
Tmed2	1.90	27.1	4.8	0.00166	0.00953	-0.99
Itgb3	1.91	30.4	8.4	0.00005	0.00125	2.63
H2-K1	1.93	28.4	6.7	0.00022	0.00274	1.13
Chpf2	1.93	25.4	3.4	0.01045	0.03337	-2.91
Nrm	1.93	25.9	3.2	0.01347	0.03975	-3.17
Plac8	1.95	31.3	4.7	0.00198	0.01071	-1.16
Slc29a1	1.95	27.0	5.2	0.00106	0.00706	-0.51
Acads	1.95	27.3	3.0	0.01793	0.04900	-3.46
Emc3	1.96	25.1	3.2	0.01444	0.04178	-3.24
Pdhb	1.97	28.9	6.3	0.00034	0.00356	0.69
Muc13	1.98	25.6	4.3	0.00316	0.01481	-1.66
Ppp1r10	1.98	25.5	4.8	0.00177	0.00993	-1.06
Slc30a5	1.98	24.9	4.9	0.00145	0.00866	-0.84
Gfer	1.98	23.9	4.5	0.00244	0.01220	-1.39
Prdx3	1.99	28.3	5.0	0.00135	0.00830	-0.76
FRRS1	2.00	27.5	6.9	0.00019	0.00263	1.25
Ank1	2.01	25.0	4.1	0.00400	0.01715	-1.91
Bri3bp	2.03	26.2	3.3	0.01184	0.03627	-3.03
Tgoln1	2.03	24.8	3.0	0.01836	0.04992	-3.48
Rab11b	2.03	32.7	12.4	0.00000	0.00045	5.15
Tspan14	2.04	28.1	5.8	0.00058	0.00505	0.11
P2rx1	2.04	24.2	4.3	0.00323	0.01494	-1.68
Gm2a	2.05	28.8	8.7	0.00004	0.00115	2.81
Glb1l	2.05	24.2	4.1	0.00397	0.01703	-1.90
Itgb2l	2.05	30.8	8.3	0.00006	0.00133	2.51
Psap	2.06	31.7	9.5	0.00002	0.00087	3.42
H2-D1	2.07	31.2	9.0	0.00003	0.00106	3.03
Rab5c	2.07	31.8	11.5	0.00001	0.00049	4.67
Slc6a4	2.08	24.7	3.7	0.00656	0.02395	-2.42
Rab1	2.08	32.1	12.2	0.00000	0.00045	5.03
Hexa	2.08	32.2	7.8	0.00009	0.00174	2.07
Got2	2.08	31.6	6.3	0.00035	0.00365	0.64

Mcemp1	2.09	29.7	8.2	0.00006	0.00136	2.44
Tlr13	2.09	24.5	3.9	0.00507	0.02007	-2.16
Pcyox1l	2.10	27.2	6.5	0.00028	0.00318	0.87
Rdh12	2.11	27.6	6.9	0.00019	0.00263	1.26
Lgals9	2.11	29.0	7.6	0.00010	0.00181	1.95
Cr1l	2.12	27.5	5.8	0.00057	0.00505	0.12
Wls	2.13	26.4	3.3	0.01291	0.03853	-3.12
Mpeg1	2.14	27.5	7.7	0.00009	0.00174	2.05
Ctsh	2.14	29.6	10.8	0.00001	0.00057	4.24
Notch2	2.14	25.2	3.9	0.00527	0.02070	-2.20
Sgsh	2.15	27.5	5.7	0.00064	0.00533	0.02
H2-Aa	2.15	25.4	4.8	0.00173	0.00980	-1.03
Lmbrd2	2.16	25.2	5.1	0.00121	0.00781	-0.66
Srsf4	2.16	29.2	5.7	0.00063	0.00530	0.03
Ssr3	2.17	26.4	3.0	0.01848	0.04999	-3.49
Nagpa	2.17	26.2	5.6	0.00069	0.00560	-0.07
Atp2a1	2.17	29.0	6.9	0.00019	0.00265	1.25
Cnpy3	2.17	24.2	3.0	0.01800	0.04914	-3.46
Siglec1	2.18	30.9	6.8	0.00021	0.00269	1.18
Pla2g15	2.18	28.1	8.5	0.00005	0.00120	2.69
Rab27b	2.18	27.8	6.8	0.00020	0.00269	1.19
Mpo	2.20	34.4	9.8	0.00002	0.00078	3.58
Mtx1	2.21	24.3	3.1	0.01691	0.04679	-3.40
Stom	2.22	30.3	13.0	0.00000	0.00042	5.43
Plrg1	2.23	28.3	7.7	0.00009	0.00176	2.03
mFLJ00021	2.23	25.2	3.2	0.01527	0.04338	-3.29
Arsa	2.25	26.1	4.2	0.00341	0.01540	-1.74
Atrn	2.26	25.0	4.7	0.00200	0.01081	-1.18
Clec2d	2.26	25.4	4.0	0.00465	0.01884	-2.07
Lman2l	2.27	24.8	3.2	0.01446	0.04180	-3.24
Tmem40	2.27	25.2	3.1	0.01774	0.04851	-3.45
Bcap31	2.28	28.5	6.5	0.00028	0.00318	0.88
Ffar2	2.28	24.8	3.7	0.00738	0.02593	-2.54
Galns	2.29	30.8	12.2	0.00000	0.00045	5.02
Pdha1	2.29	27.8	5.4	0.00093	0.00656	-0.37
Prpf38b	2.29	26.7	3.7	0.00741	0.02596	-2.54
Fut8	2.30	26.9	5.5	0.00077	0.00590	-0.18
Hrg	2.30	24.7	4.5	0.00260	0.01271	-1.46
Nceh1	2.30	25.4	4.1	0.00386	0.01671	-1.87
Rmcs1	2.31	26.9	3.3	0.01233	0.03725	-3.07
F5	2.31	27.9	8.8	0.00004	0.00112	2.93
Ctbs	2.32	28.7	8.9	0.00003	0.00108	3.00
Syne3	2.33	25.7	3.3	0.01333	0.03952	-3.15
Dpep1	2.34	26.3	4.8	0.00174	0.00983	-1.03
Jup	2.35	28.1	3.3	0.01221	0.03717	-3.06
Rab37	2.35	25.3	3.2	0.01422	0.04144	-3.22
Isg15	2.36	29.0	6.0	0.00045	0.00426	0.38
Ca2	2.36	31.8	6.2	0.00038	0.00378	0.57
Arglu1	2.36	28.7	7.6	0.00010	0.00187	1.90
Cd1d1	2.38	25.3	3.6	0.00775	0.02682	-2.59
Gp1bb	2.38	29.5	12.1	0.00000	0.00045	4.98
Rab6a	2.38	25.3	3.5	0.00912	0.03015	-2.76
Prg3	2.40	25.3	3.5	0.00972	0.03171	-2.82
Pilrb2	2.40	24.2	4.3	0.00316	0.01481	-1.66
Agrn	2.40	27.2	4.4	0.00296	0.01413	-1.58
Ivd	2.41	25.1	3.5	0.00907	0.03005	-2.75
Sacm1l	2.43	26.9	5.1	0.00125	0.00791	-0.68
Col1a2	2.44	29.2	6.9	0.00020	0.00268	1.21
Ca1	2.46	30.0	7.1	0.00017	0.00246	1.41
Cisd2	2.47	24.9	3.5	0.00928	0.03054	-2.77
Il1rap	2.47	24.8	3.6	0.00821	0.02803	-2.65
St3gal2	2.48	23.8	6.6	0.00024	0.00291	1.02
Naglu	2.49	29.7	7.5	0.00012	0.00197	1.77
Hbbt1	2.50	36.8	7.7	0.00010	0.00183	1.94

Aga	2.50	27.7	7.8	0.00009	0.00174	2.06
Plbd2	2.50	28.9	12.0	0.00000	0.00045	4.95
Txnrd2	2.50	25.2	3.3	0.01339	0.03960	-3.15
Lipa	2.51	26.5	3.9	0.00543	0.02120	-2.22
Triobp	2.51	24.1	4.0	0.00466	0.01884	-2.06
Fth1	2.53	31.9	13.3	0.00000	0.00038	5.58
Ly6c2	2.53	30.8	3.3	0.01329	0.03946	-3.15
P2rx4	2.54	25.6	4.1	0.00415	0.01759	-1.94
Renbp	2.54	26.8	6.5	0.00026	0.00310	0.92
Col11a2	2.54	24.8	3.6	0.00851	0.02887	-2.68
Abhd16a	2.56	25.0	3.9	0.00563	0.02165	-2.25
Ppbp	2.56	29.0	6.1	0.00042	0.00402	0.45
Tgfb1	2.56	27.8	8.6	0.00004	0.00116	2.75
Cldn1	2.58	25.1	3.2	0.01372	0.04040	-3.18
Ctsg	2.59	30.1	11.8	0.00000	0.00047	4.83
Mitd1	2.59	24.7	4.5	0.00247	0.01229	-1.40
Hexb	2.60	33.4	9.6	0.00002	0.00087	3.38
Asah1	2.60	31.2	12.6	0.00000	0.00045	5.25
Tf	2.60	25.7	3.3	0.01248	0.03757	-3.08
Adcy7	2.61	24.1	6.5	0.00027	0.00312	0.91
Hdhd2	2.63	26.7	4.0	0.00487	0.01942	-2.10
Vdac2	2.64	31.5	8.2	0.00006	0.00144	2.37
Col9a1	2.65	25.2	3.3	0.01247	0.03756	-3.08
Slc14a1	2.66	29.1	7.9	0.00008	0.00172	2.10
Fabp4	2.68	29.4	5.9	0.00051	0.00469	0.25
Thbs1	2.68	32.1	10.2	0.00002	0.00073	3.78
Rab24	2.68	26.7	7.7	0.00009	0.00177	2.01
Ctsc	2.69	29.4	4.4	0.00294	0.01405	-1.57
Chi3l1	2.69	31.5	10.6	0.00001	0.00062	4.07
Mgst3	2.69	25.1	4.6	0.00215	0.01141	-1.26
Far1	2.70	26.0	5.3	0.00092	0.00652	-0.37
Endod1	2.72	26.0	3.1	0.01573	0.04431	-3.32
Btd	2.72	24.7	6.9	0.00019	0.00258	1.28
Treml1	2.73	28.9	7.1	0.00016	0.00243	1.43
Vwf	2.82	29.7	9.6	0.00002	0.00087	3.39
Gnaz	2.84	25.0	5.0	0.00142	0.00855	-0.82
Vdac3	2.86	29.8	4.1	0.00421	0.01773	-1.95
Creg1	2.87	29.5	8.3	0.00006	0.00136	2.45
Mmp9	2.87	33.0	11.8	0.00001	0.00048	4.73
Ctsb	2.88	31.7	14.8	0.00000	0.00038	6.21
Grn	2.89	27.1	3.6	0.00791	0.02725	-2.61
Camp	2.90	32.3	10.2	0.00001	0.00072	3.81
Igh	2.92	26.4	4.7	0.00202	0.01089	-1.18
Vdac1	2.92	31.3	9.2	0.00003	0.00104	3.10
Nipsnap3b	2.93	27.0	5.4	0.00094	0.00658	-0.38
Sppl2a	2.94	25.4	5.1	0.00120	0.00776	-0.65
Pld4	2.95	30.4	11.0	0.00001	0.00057	4.29
Lilrb4	2.95	25.8	4.2	0.00368	0.01623	-1.81
HBB1	2.95	29.2	6.3	0.00034	0.00356	0.69
H2-Q10	2.96	25.1	6.0	0.00044	0.00414	0.40
Ech1	2.96	26.8	6.9	0.00018	0.00252	1.32
Ppic	2.96	27.2	9.3	0.00003	0.00094	3.27
Tomm40	2.97	26.6	5.6	0.00071	0.00567	-0.10
Ngp	3.00	36.3	9.8	0.00002	0.00083	3.51
Ltf	3.01	36.1	16.5	0.00000	0.00026	6.82
Aqp1	3.02	28.2	8.3	0.00006	0.00135	2.48
Epdr1	3.03	27.8	11.4	0.00001	0.00051	4.61
Acan	3.04	24.6	4.9	0.00151	0.00893	-0.88
Atp6v0d2	3.04	24.6	4.8	0.00173	0.00983	-1.02
Prcp	3.08	27.1	6.6	0.00026	0.00310	0.95
Chil3	3.08	37.0	13.1	0.00000	0.00042	5.37
Ftl1	3.09	33.6	14.3	0.00000	0.00038	6.01
Cd300lf	3.09	25.6	6.5	0.00027	0.00312	0.90
Aldh1l2	3.13	24.5	3.5	0.00926	0.03052	-2.77

Siae	3.14	26.7	8.5	0.00005	0.00124	2.64
Cyb5r3	3.14	27.4	3.4	0.01136	0.03511	-2.98
Cnp	3.15	25.4	4.8	0.00172	0.00980	-1.02
Mfsd2b	3.16	25.6	7.6	0.00010	0.00181	1.95
Cd177	3.16	34.4	7.5	0.00011	0.00194	1.82
Gla	3.17	26.8	3.4	0.01132	0.03505	-2.98
Ssbp1	3.20	26.7	3.8	0.00637	0.02348	-2.38
Mybpc1	3.21	25.0	5.8	0.00053	0.00485	0.19
Ear2	3.22	30.5	9.0	0.00004	0.00111	2.96
Engase	3.22	25.0	6.8	0.00021	0.00269	1.16
Cst3	3.24	29.2	13.3	0.00000	0.00038	5.58
Ctss	3.24	29.9	9.2	0.00003	0.00104	3.09
Ttyh3	3.25	25.1	4.9	0.00159	0.00923	-0.93
Plg	3.26	25.2	5.5	0.00078	0.00599	-0.20
Gpr56	3.29	25.1	5.9	0.00050	0.00459	0.26
Samm50	3.31	26.5	4.5	0.00264	0.01281	-1.46
Tmem33	3.31	25.2	4.1	0.00447	0.01842	-2.01
Lcn2	3.32	34.6	15.1	0.00000	0.00038	6.31
Irgm1	3.33	24.5	4.2	0.00358	0.01590	-1.78
Olfm4	3.34	31.4	11.0	0.00001	0.00057	4.31
Angpt1	3.35	24.6	6.5	0.00026	0.00310	0.93
Ifggd1	3.38	28.1	9.9	0.00002	0.00076	3.64
Acp5	3.38	30.1	9.4	0.00003	0.00095	3.25
H2-T24	3.41	23.9	7.4	0.00011	0.00194	1.79
Fgb	3.44	31.3	9.7	0.00002	0.00084	3.48
Ccl6	3.45	25.6	5.7	0.00065	0.00540	-0.01
C3	3.48	34.0	17.0	0.00000	0.00025	6.99
Ctsk	3.49	25.2	4.9	0.00158	0.00921	-0.93
Cpq	3.53	29.3	10.5	0.00001	0.00065	4.01
Ctsl	3.54	25.7	6.2	0.00038	0.00378	0.56
Cd163	3.56	26.6	6.4	0.00031	0.00347	0.75
Tcn2	3.57	28.8	12.9	0.00000	0.00042	5.36
Serpine2	3.60	25.8	6.7	0.00023	0.00282	1.07
Bst2	3.62	25.4	4.0	0.00503	0.01993	-2.14
Itgad	3.64	25.9	7.3	0.00013	0.00212	1.64
Fga	3.67	31.3	8.4	0.00005	0.00130	2.55
Chit1	3.71	25.9	5.1	0.00126	0.00792	-0.68
Lnp	3.73	25.8	6.5	0.00026	0.00310	0.92
Itih5	3.73	25.0	8.2	0.00006	0.00135	2.48
Pttg1ip	3.85	26.4	5.4	0.00086	0.00623	-0.28
Ighg	3.87	25.5	6.0	0.00045	0.00426	0.37
F11r	3.88	25.5	7.7	0.00009	0.00177	2.01
Retnlg	3.91	30.8	4.1	0.00434	0.01819	-1.98
Tmem50b	3.92	24.3	10.1	0.00001	0.00072	3.81
Lyz2	3.96	35.0	10.0	0.00002	0.00076	3.66
Fcgr4	3.98	24.5	6.7	0.00023	0.00282	1.08
Lrg1	3.99	29.8	13.9	0.00000	0.00038	5.75
Lgals3bp	4.02	30.1	12.2	0.00000	0.00045	4.95
Mtch2	4.07	27.3	4.1	0.00440	0.01832	-2.00
Adam8	4.09	27.0	5.3	0.00105	0.00706	-0.50
Aoah	4.17	25.5	9.5	0.00002	0.00086	3.44
Fgg	4.22	30.6	11.2	0.00001	0.00057	4.38
Timp2	4.28	25.9	4.9	0.00162	0.00934	-0.95
Pros1	4.32	25.2	8.0	0.00007	0.00152	2.28
Mmp8	4.33	31.5	22.0	0.00000	0.00007	8.27
Elane	4.40	32.4	24.2	0.00000	0.00007	8.67
Orm1	4.41	25.3	8.2	0.00006	0.00136	2.45
Prtn3	4.49	32.5	20.3	0.00000	0.00009	7.89
Pglyrp1	4.51	31.6	3.6	0.00863	0.02911	-2.70
Adarb2	4.66	26.9	6.2	0.00040	0.00390	0.50
Dpp4	4.66	26.2	5.5	0.00082	0.00611	-0.24
Hp	4.68	32.0	22.0	0.00000	0.00007	8.25
Abca13	4.84	25.1	10.6	0.00001	0.00060	4.13
Mcpt8	4.99	26.3	14.3	0.00000	0.00038	6.02

Qsox1	4.99	29.2	13.7	0.00000	0.00038	5.62
Nbeal2	5.02	26.5	6.2	0.00039	0.00380	0.54
Ly6g6f	5.04	25.4	8.7	0.00004	0.00113	2.85
Clec1b	5.16	25.3	12.1	0.00000	0.00045	4.96
Chil4	5.19	27.9	3.8	0.00634	0.02348	-2.38
Olr1	5.24	26.3	5.4	0.00087	0.00627	-0.30
Ggh	5.25	27.4	10.9	0.00001	0.00057	4.22
Crispld2	5.32	27.1	4.6	0.00236	0.01196	-1.34
Ear6	5.44	28.3	5.0	0.00138	0.00840	-0.78
Actb	5.85	29.0	11.0	0.00001	0.00057	4.26
Actb	5.98	27.2	6.8	0.00022	0.00274	1.15
Cfp	6.19	26.9	10.0	0.00002	0.00076	3.64
Hbb-b2	6.50	26.6	11.1	0.00001	0.00057	4.34
Prss34	7.55	27.9	14.3	0.00000	0.00038	5.88

Supplemental Table 2. Differentially expressed genes between *Nbeal2+/+* and *Nbeal2-/-* bone marrow neutrophils

Affy ID	logFC (log2)	AveExpr	t	P.Value	adj.P.Val	B	SYMBOL
**17284493**	**-3.24**	**5.6**	**-6.18**	**0.000124274**	**0.042**	**1.55**	**Igh-VJ558**
**17467377**	**-2.15**	**5.8**	**-8.05**	**1.43E-05**	**0.014**	**3.47**	**Igkv9-124**
**17284648**	**-1.57**	**10.6**	**-8.96**	**5.70E-06**	**0.009**	**4.24**	**Ighm**
**17439367**	**-1.32**	**8.0**	**-11.32**	**7.24E-07**	**0.004**	**5.84**	**Cxcl13**
**17284432**	**-1.25**	**9.3**	**-8.74**	**7.06E-06**	**0.009**	**4.06**	**Igk-V1**
**17284356**	**-0.95**	**12.6**	**-8.92**	**5.96E-06**	**0.009**	**4.20**	**Igh-VJ558**
**17472192**	**-0.90**	**10.1**	**-6.55**	**7.81E-05**	**0.034**	**1.97**	**Mgp**
**17284314**	**-0.81**	**11.5**	**-7.47**	**2.67E-05**	**0.020**	**2.93**	**Igh-VJ558**
**17284605**	**-0.74**	**11.1**	**-6.24**	**0.000115466**	**0.042**	**1.61**	**Ighm**
**17335770**	**-0.73**	**10.2**	**-7.59**	**2.35E-05**	**0.018**	**3.04**	**Abcg1**
**17219139**	**-0.69**	**8.0**	**-7.31**	**3.22E-05**	**0.021**	**2.76**	**Rgs5**
**17213336**	**-0.64**	**9.1**	**-6.22**	**0.000117513**	**0.042**	**1.60**	**Bmpr2**
17295233	0.60	9.9	6.33	0.000102145	0.040	1.73	Hmgcr
17387426	0.61	7.2	6.24	0.00011481	0.042	1.62	Gm13710
17412374	0.61	8.3	7.33	3.14E-05	0.021	2.78	Bach2
17445308	0.62	6.9	7.28	3.33E-05	0.022	2.73	Cyp51
17356216	0.62	8.1	6.50	8.29E-05	0.036	1.92	Pcx
17215788	0.63	9.8	6.21	0.000119108	0.042	1.58	Asb1
17265386	0.66	7.4	6.78	5.92E-05	0.028	2.22	Scimp
17466119	0.66	10.2	6.66	6.85E-05	0.032	2.09	Mkrn1
17489320	0.69	8.1	7.00	4.55E-05	0.026	2.46	Cd22
17219789	0.69	8.6	6.31	0.000105866	0.040	1.69	Kmo
17423395	0.70	7.6	6.21	0.00011977	0.042	1.58	Slc26a7
17334449	0.71	9.9	6.55	7.85E-05	0.034	1.97	Hagh
17293348	0.72	7.0	6.45	8.84E-05	0.038	1.86	Ctla2b
17462602	0.73	4.8	6.77	6.00E-05	0.028	2.21	Gm10319
17270837	0.73	10.2	6.37	9.75E-05	0.040	1.77	Cd79b
17500391	0.74	8.3	8.19	1.24E-05	0.013	3.59	Rnf122
17307588	0.74	9.3	9.71	2.83E-06	0.005	4.80	Fdft1
17277685	0.75	7.0	7.16	3.79E-05	0.023	2.62	Oog1
17282025	0.76	11.0	6.23	0.000117028	0.042	1.60	Sptb
17281312	0.76	8.2	6.92	5.01E-05	0.026	2.37	Slc25a21
17430729	0.76	10.9	7.14	3.91E-05	0.023	2.59	Epb4.1
17475127	0.78	8.9	7.17	3.77E-05	0.023	2.62	Cd79a
17520560	0.78	9.9	6.15	0.000129273	0.043	1.51	Tfdp2
17313504	0.80	10.5	9.00	5.52E-06	0.009	4.27	Srebf2
17268404	0.81	9.9	6.58	7.53E-05	0.034	2.00	Pnpo
17290173	0.81	8.6	10.67	1.23E-06	0.004	5.45	Hmgcs1
17314888	0.82	8.0	6.21	0.000120085	0.042	1.58	Gpd1
17354995	0.82	11.4	6.86	5.37E-05	0.027	2.31	Fech
17416325	0.83	9.0	7.05	4.33E-05	0.025	2.50	Dhcr24
17311807	0.84	7.7	6.35	0.000100294	0.040	1.74	Sqle
17278345	0.86	11.1	7.56	2.41E-05	0.018	3.02	Glrx5
17408414	0.87	10.9	7.93	1.63E-05	0.015	3.36	Fam46c
17372604	0.88	11.2	7.59	2.35E-05	0.018	3.04	Ube2l6

17229466	0.89	8.2	7.72	2.03E-05	0.017	3.17	Hsd17b7
17285056	0.90	8.1	9.68	2.92E-06	0.005	4.78	Idi1
17435584	0.97	9.2	11.53	6.13E-07	0.004	5.96	Insig1
17467250	0.97	12.0	7.32	3.18E-05	0.021	2.77	Snca
17290190	1.00	5.5	6.90	5.16E-05	0.026	2.34	D13Ertd608e
17358103	1.03	9.8	7.73	2.02E-05	0.017	3.17	Aldh1a1
17538575	1.05	12.1	8.83	6.49E-06	0.009	4.13	Apex2
17495821	1.08	10.2	7.16	3.80E-05	0.023	2.62	Cdr2
17253476	1.09	8.3	8.89	6.13E-06	0.009	4.18	NA
17362922	1.09	7.3	10.49	1.43E-06	0.004	5.33	Ms4a1
17490274	1.14	8.5	10.31	1.67E-06	0.004	5.22	Spib
17531581	1.18	8.4	8.44	9.59E-06	0.012	3.81	Nradd
17266967	1.19	11.2	7.98	1.53E-05	0.014	3.41	Ccl3
17515315	1.22	9.4	9.32	4.05E-06	0.007	4.52	Ldlr
17428473	1.28	7.9	6.96	4.78E-05	0.026	2.41	Gm12839
17457171	1.29	10.0	8.27	1.14E-05	0.012	3.66	Bpgm
17507184	1.30	6.9	10.64	1.26E-06	0.004	5.43	Cd209d
17478511	1.39	8.4	9.90	2.39E-06	0.005	4.94	Siglech
17444364	1.42	9.5	8.02	1.49E-05	0.014	3.44	Fam220a
17374488	1.49	11.7	10.90	1.02E-06	0.004	5.59	**Thbs1**
17248282	1.52	8.7	6.32	0.000103998	0.040	1.71	Hbq1a
17423577	1.65	6.6	6.93	4.95E-05	0.026	2.38	Atp6v0d2
17546219	2.06	6.9	10.34	1.63E-06	0.004	5.23	NA
17458439	2.16	8.1	8.40	9.95E-06	0.012	3.78	Gpnmb
17546215	2.24	6.3	9.19	4.61E-06	0.008	4.41	Mid1
17477391	2.61	7.3	10.26	1.75E-06	0.004	5.18	Klk1
17546762	2.77	9.6	17.20	1.57E-08	0.000	8.24	Mid1
17531591	3.77	9.8	30.23	7.65E-11	0.000	10.24	Nbeal2

Supplemental Table 3 . Gene Ontology cellular compartment terms enriched in 2.5-fold downregulated proteins in *Nbeal2-/-* neutrophils

GO cellular component complete	Mus musculus - REFLIST (22322)	2.5 Fold Downregulated	Expected	Fold Enrichment	Bon Ferroni corrected P-value
fibrinogen complex (GO:0005577)	7	4	0.04	89.2	1.94E-04
extrinsic component of external side of plasma membrane (GO:0031232)	7	3	0.04	66.9	1.77E-02
platelet alpha granule (GO:0031091)	17	6	0.11	55.09	2.37E-06
specific granule (GO:0042581)	13	4	0.08	48.03	2.24E-03
pore complex (GO:0046930)	15	4	0.1	41.63	3.94E-03
blood microparticle (GO:0072562)	126	12	0.81	14.87	6.26E-08
lytic vacuole (GO:0000323)	442	27	2.83	9.54	1.43E-15
lysosome (GO:0005764)	442	27	2.83	9.54	1.43E-15
secretory granule (GO:0030141)	319	19	2.04	9.3	4.27E-10
vacuole (GO:0005773)	520	28	3.33	8.41	7.59E-15
mitochondrial outer membrane (GO:0005741)	152	8	0.97	8.22	9.06E-03
secretory vesicle (GO:0099503)	453	22	2.9	7.58	2.86E-10
organelle outer membrane (GO:0031968)	167	8	1.07	7.48	1.77E-02
outer membrane (GO:0019867)	171	8	1.1	7.3	2.10E-02
extracellular space (GO:0005615)	1435	58	9.19	6.31	2.11E-28
extracellular matrix (GO:0031012)	480	19	3.07	6.18	4.25E-07
proteinaceous extracellular matrix (GO:0005578)	340	13	2.18	5.97	4.49E-04
external side of plasma membrane (GO:0009897)	324	12	2.08	5.78	1.79E-03
extracellular vesicle (GO:1903561)	2560	83	16.4	5.06	9.38E-38
extracellular organelle (GO:0043230)	2566	83	16.44	5.05	1.12E-37
extracellular exosome (GO:0070062)	2545	82	16.3	5.03	6.39E-37
apical part of cell (GO:0045177)	381	12	2.44	4.92	9.16E-03
cell surface (GO:0009986)	831	24	5.32	4.51	9.33E-07

side of membrane (GO:0098552)	499	14	3.2	4.38	5.78E-03
extracellular region part (GO:0044421)	3698	100	23.69	4.22	3.23E-42
extracellular region (GO:0005576)	4224	106	27.06	3.92	3.24E-43
vesicle (GO:0031982)	3758	92	24.07	3.82	1.50E-33
intracellular vesicle (GO:0097708)	1551	36	9.94	3.62	1.22E-08
cytoplasmic vesicle (GO:0031410)	1523	35	9.76	3.59	3.36E-08
bounding membrane of organelle (GO:0098588)	925	18	5.93	3.04	3.61E-02
endomembrane system (GO:0012505)	3445	47	22.07	2.13	2.16E-04
cytoplasmic part (GO:0044444)	6634	83	42.5	1.95	2.46E-09
membrane-bounded organelle (GO:0043227)	11210	111	71.81	1.55	1.83E-08
organelle (GO:0043226)	12090	112	77.45	1.45	2.04E-06
cytoplasm (GO:0005737)	10007	91	64.11	1.42	5.81E-03
cell part (GO:0044464)	14857	117	95.18	1.23	4.60E-02
cell (GO:0005623)	14859	117	95.19	1.23	4.64E-02
cellular_component (GO:0005575)	20700	143	132.61	1.08	2.57E-02
Analysis Type:	PANTHER Overrepresentation Test (release 20160715)				
Annotation Version and Release Date:	GO Ontology database Released 2016-10-27				

References

1. Guerrero JA, Bennett C, van der Weyden L, et al. Gray platelet syndrome: proinflammatory megakaryocytes and alpha-granule loss cause myelofibrosis and confer metastasis resistance in mice. *Blood.* 2014;124(24):3624-35.
2. Lyons PA, Koukoulaki M, Hatton A, et al. Microarray analysis of human leucocyte subsets: the advantages of positive selection and rapid purification. *BMC Genomics.* 2007;8(64.
3. Stacey MA, Marsden M, Pham NT, et al. Neutrophils recruited by IL-22 in peripheral tissues function as TRAIL-dependent antiviral effectors against MCMV. *Cell Host Microbe.* 2014;15(4):471-83.
4. Speak AO, Te Vruchte D, Davis LC, et al. Altered distribution and function of natural killer cells in murine and human Niemann-Pick disease type C1. *Blood.* 2014;123(1):51-60.
5. White JK, Gerdin AK, Karp NA, et al. Genome-wide generation and systematic phenotyping of knockout mice reveals new roles for many genes. *Cell.* 2013;154(2):452-64.
6. von Kockritz-Blickwede M, Rohde M, Oehmcke S, et al. Immunological mechanisms underlying the genetic predisposition to severe Staphylococcus aureus infection in the mouse model. *Am J Pathol.* 2008;173(6):1657-68.
7. Stacey MA, Clare S, Clement M, et al. The antiviral restriction factor IFN-induced transmembrane protein 3 prevents cytokine-driven CMV pathogenesis. *J Clin Invest.* 2017;127(4):1463-74.
8. Rorvig S, Ostergaard O, Heegaard NH, and Borregaard N. Proteome profiling of human neutrophil granule subsets, secretory vesicles, and cell membrane: correlation with transcriptome profiling of neutrophil precursors. *J Leukoc Biol.* 2013;94(4):711-21.
9. Daley JM, Thomay AA, Connolly MD, et al. Use of Ly6G-specific monoclonal antibody to deplete neutrophils in mice. *J Leukoc Biol.* 2008;83(1):64-70.

Full unedited gel for Supplementary Figure 5

Supplementary Figure 5B

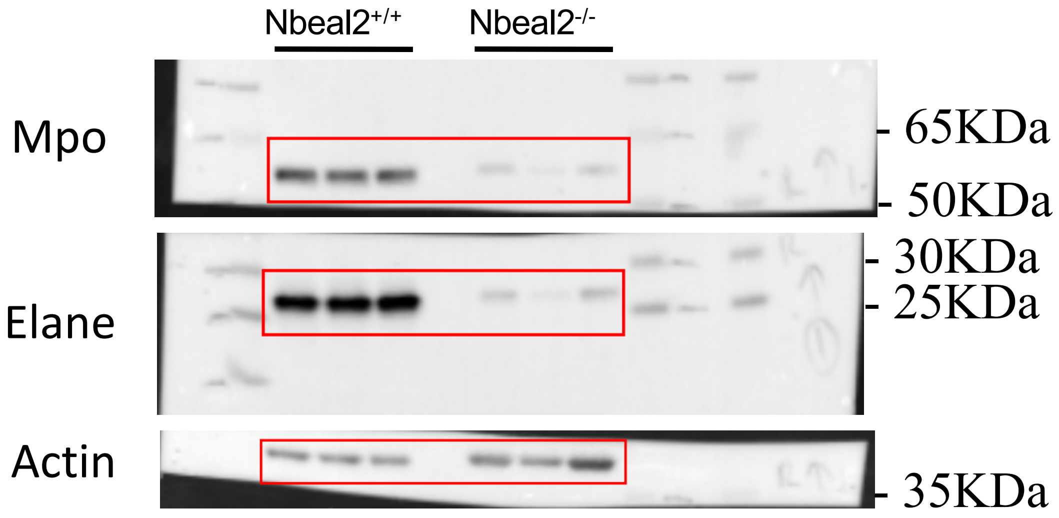

Supplementary Figure 5C

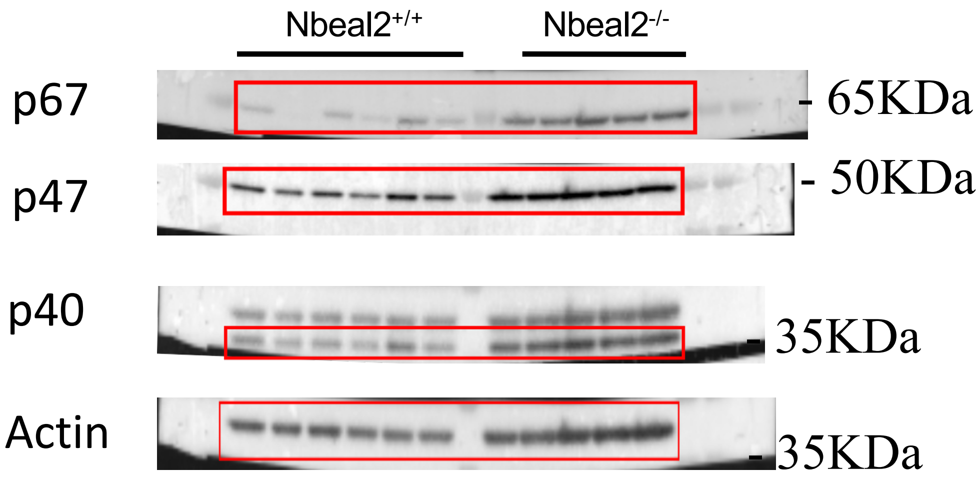

Supplement: Supplemental data [file jci-127-91684-s001.pdf]
